# Distinct functions of chemokine receptor axes in the atherogenic mobilization and recruitment of classical monocytes

**DOI:** 10.1002/emmm.201201717

**Published:** 2013-02-18

**Authors:** Oliver Soehnlein, Maik Drechsler, Yvonne Döring, Dirk Lievens, Helene Hartwig, Klaus Kemmerich, Almudena Ortega-Gómez, Manuela Mandl, Santosh Vijayan, Delia Projahn, Christoph D Garlichs, Rory R Koenen, Mihail Hristov, Esther Lutgens, Alma Zernecke, Christian Weber

**Affiliations:** 1 Institute for Cardiovascular Prevention, Ludwig-Maximilians-UniversityMunich, Germany; 2 Department of Pathology, Academic Medical Center Amsterdam, University of Amsterdamthe Netherlands; 3 Institute for Molecular Cardiovascular Research (IMCAR), RWTH Aachen UniversityGermany; 4 Department of Cardiology, Friedrich-Alexander University ErlangenGermany; 5 Cardiovascular Research Institute Maastricht, Maastricht UniversityThe Netherlands; 6 Department of Medical Biochemistry, Academic Medical Center Amsterdam, University of Amsterdamthe Netherlands; 7 Rudolf-Virchow-Center/DFG Research Center for Experimental Biomedicine, University of WürzburgGermany; 8 DZHK (German Centre for Cardiovascular Research), partner site Munich Heart AllianceMunich, Germany; 9 Present address: Department of Vascular Surgery, Technical University MunichGermany

**Keywords:** atherosclerosis, chemokine, mobilization, monocyte, recruitment

## Abstract

We used a novel approach of cytostatically induced leucocyte depletion and subsequent reconstitution with leucocytes deprived of classical (inflammatory/Gr1^hi^) or non-classical (resident/Gr1^lo^) monocytes to dissect their differential role in atheroprogression under high-fat diet (HFD). Apolipoprotein E-deficient (*Apoe*^*−/−*^) mice lacking classical but not non-classical monocytes displayed reduced lesion size and macrophage and apoptotic cell content. Conversely, HFD induced a selective expansion of classical monocytes in blood and bone marrow. Increased CXCL1 levels accompanied by higher expression of its receptor CXCR2 on classical monocytes and inhibition of monocytosis by CXCL1-neutralization indicated a preferential role for the CXCL1/CXCR2 axis in mobilizing classical monocytes during hypercholesterolemia. Studies correlating circulating and lesional classical monocytes in gene-deficient *Apoe*^*−/−*^ mice, adoptive transfer of gene-deficient cells and pharmacological modulation during intravital microscopy of the carotid artery revealed a crucial function of CCR1 and CCR5 but not CCR2 or CX_3_CR1 in classical monocyte recruitment to atherosclerotic vessels. Collectively, these data establish the impact of classical monocytes on atheroprogression, identify a sequential role of CXCL1 in their mobilization and CCR1/CCR5 in their recruitment.

## INTRODUCTION

Monocytes and their descendants are the most abundant leucocytes in atherosclerotic lesions (Weber et al, 2008). Studies correlating systemic monocyte counts with severity of atherosclerosis in humans and mice suggest a role of monocytes in disease progression (Olivares et al, 1993; Swirski et al, 2006). Depletion strategies have provided evidence for the global significance of monocytes in atheroprogression (Ylitalo et al, 1994), with more recent work indicating a stage-specific influence, whereby monocytes promote atherosclerosis at early stages but not at later time points (Stoneman et al, 2007). With the emergence of at least two functionally different monocyte subsets in humans and mice termed classical (inflammatory, Gr1^hi^) and non-classical (resident, Gr1^lo^) monocytes (Geissmann et al, 2003), it remains to be determined, which differential impact they have on atherosclerosis.

Hypercholesterolemia selectively increases circulating classical monocyte counts (Swirski et al, 2007) and induces phenotypic changes favoring emigration into atherosclerotic lesions (Soehnlein et al, 2009), suggesting a prominent role of classical monocytes in atherosclerosis. Fundamental to the importance of monocytes in atherosclerosis is their accumulation within atherosclerotic lesions (Weber et al, 2008) a process regulated at various levels, *i.e.* mobilization from sites of production, recruitment, and survival in the lesion (Gautier et al, 2009). Extravasation of monocytes requires the coordinated interaction of selectins, adhesion molecules, and chemokines (Imhof & Aurrand-Lions, 2004). With the discovery of monocyte subsets a concept has emerged, wherein the relative expression of adhesion molecules or chemokine receptors governs their recruitment behaviour. In this context, it has been suggested that classical monocytes, which express higher levels of CCR2 compared to non-classical monocytes (Weber et al, 2000), are recruited to the site of inflammation in *Ccr2*^*−/−*^ mice in lower numbers (Boring et al, 1998). In addition, chemokines and their receptors fulfill important roles in monocyte mobilization from the bone marrow (BM). Accordingly, as CCR2 is essential in mobilization of classical monocytes from the BM, these mice also exhibit markedly reduced numbers of circulating monocytes (Serbina & Pamer, 2006). Beyond recruitment and mobilization, chemokine receptor axes can crucially affect monocyte life span. For instance, absence of CX_3_CR1 results in reduction of non-classical monocytes blood counts, which are restored by introduction of a Bcl2 transgene, suggesting that the CX_3_C axis provides a survival signal (Landsman et al, 2009).

Thus, the objective of this study was to dissect a differential role of monocyte subsets in early stages of atherosclerosis, to clarify the chemokine-driven mechanisms underlying hypercholesterolemia-induced monocytosis, and to examine the functional involvement of chemokine receptor axes in the recruitment pattern of these monocytes independently of their homeostatic influence.

## RESULTS

### Classical monocytes drive atheroprogression

**Figure 1:**
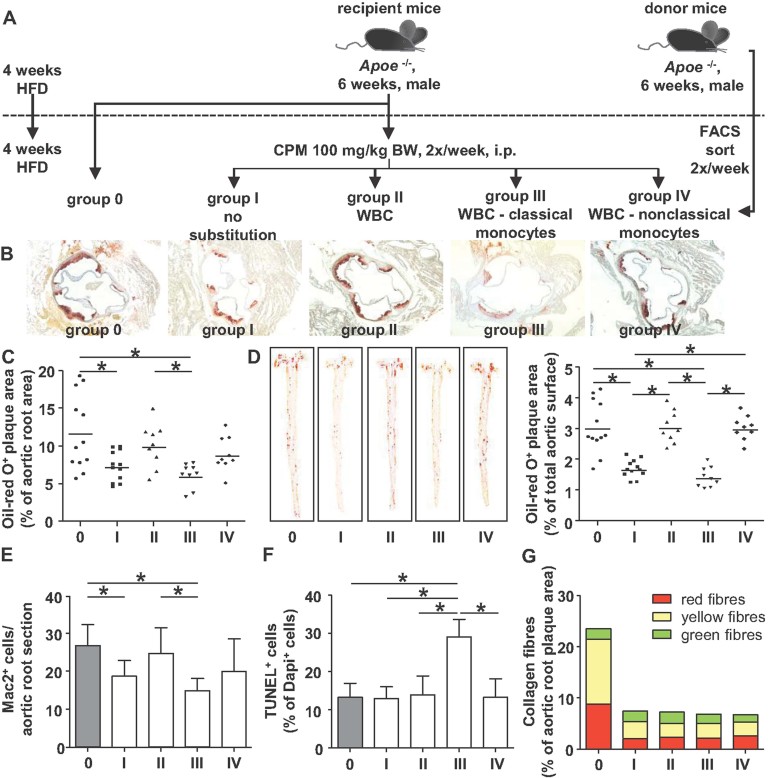
Classical monocytes are decisive during atheroprogression **A.** Scheme illustrating the approach to investigate the individual contribution of monocyte subsets to atherosclerotic lesion formation. Male *Apoe*^*−/−*^mice 6 weeks of age were fed a HFD for a total of 8 weeks. After 4 weeks of HFD, groups I to IV were treated with i.p. CPM 2×/week. Groups II to IV were reconstituted with leucocytes from *Apoe*^*−/−*^ donor mice by i.v. injections 2×/week using one donor mouse/recipient each 1 day after CPM treatment. Group II was substituted with all CD45^+^leucocytes, group III received CD45^+^ leucocytes without classical monocytes, and group IV was reconstituted with CD45^+^ leucocytes without non-classical monocytes, in each case depleted by sorting for classical *versus* non-classical monocytes.**B–D.** Assessment of atherosclerotic lesion size after staining aortic root sections (**B,C**) or aortas (**D**) with oil-red-O. Representative images and quantification are displayed.**E.** Quantification of monocyte/macrophage content in aortic roots following Mac2 staining.**F.** Quantification of TUNEL^+^ apoptotic cells.**G.** Analysis of collagen content by sirius red staining and analysis under polarized light. All data are expressed as mean ± SD. *Denotes significant differences between groups. *n* = 9 − 12 for each group (One-way ANOVA with Newman–Keuls *post hoc* test).

As selective depletion of monocyte subsets is not feasible we developed a novel approach to dissect the specific contribution of monocyte subsets to atherosclerosis (Fig 1A). In *Apoe*^*−/−*^, mice fed a high-fat diet (HFD) for 4 weeks, the mobilization of leucocytes from the BM was abrogated by application of the cytostatic drug cyclophosphamide (CPM) during subsequent 4 weeks of HFD. This resulted in an absolute leucopenia (Supporting Information Table 1). To address a specific role of monocyte subsets, mice were repeatedly reconstituted with white blood cells from age-matched donor mice, in which either classical or non-classical monocytes were selectively removed by fluorescence activated cell sorting (FACS). Lipid levels, body and spleen weight were not influenced by this regimen (Supporting Information Table 2 and 3). In addition, antibody-based depletion of monocyte subsets did not alter white blood cell activation, as assessed by analysis of CD11b expression, CD62L shedding, Annexin V-binding, and reactive oxygen species production (Supporting Information Fig 1). To further validate the transfer efficacy of the reconstitution protocol, we employed the CD45.1/CD45.2 system. The results demonstrate that this protocol allowed for virtual reconstitution of white blood cell subsets (Supporting Information Fig 2) and enabled prominent lesional accumulation of donor leucocytes (Supporting Information Fig 3).

After 8 weeks of HFD, *Apoe*^*−/−*^ mice displayed atherosclerotic lesion formation in the aortic root and the thoracic aorta which was significantly diminished by application of CPM for 4 weeks (Fig 1B–D). While reconstitution of leucopenic mice with whole white blood cells from donor mice restored lesion sizes, removal of classical monocytes from donor leucocytes but not non-classical monocytes reduced atherosclerotic lesion area to levels observed in mice receiving CPM only (Fig 1B–D). Immunohistochemical analysis of Mac2^+^ macrophage content in atherosclerotic lesions of the aortic root followed a similar pattern (Fig 1E). When analysing the number of apoptotic TUNEL^+^ cells in aortic roots, half of which originated from macrophages (Supporting Information Fig 4), we found a significant increase in mice reconstituted with leucocytes depleted of classical monocytes (Fig 1F), which are known to display higher phagocytic capacity and thereby contribute to the clearance of apoptotic cells. Analysis of collagen content by sirius red staining revealed a reduction in aortic collagen content in CPM-treated groups, likely due to decreased collagen synthesis in response to CPM (Hansen & Lorenzen, 1977). In addition, no differences within the CPM-treated groups were identified (Fig 1G). Taken together, these data reveal a dominant role of classical monocytes in atheroprogression.

### mCXCL1 mediates hypercholesterolemia-induced monocytosis

**Figure 2:**
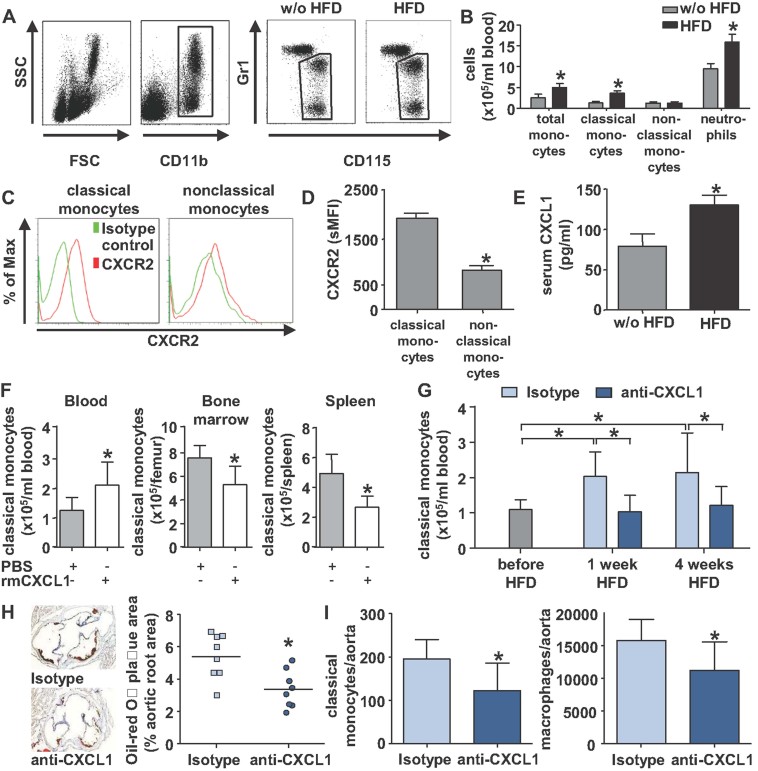
CXCL1 mediates hypercholesterolemia-induced expansion of classical monocytes **A.** *Apoe*^*−/−*^mice were fed normal chow or HFD for 8 weeks. Monocyte subsets were identified using antibodies to CD45, CD11b, CD115, and Gr1.**B.** Quantification of total monocyte counts, absolute counts of classical and non-classical monocytes, and neutrophils in the blood of *Apoe*^*−/−*^ mice fed normal chow or HFD for 8 weeks.**C.** Histograms depict specific fluorescence intensities of circulating monocyte subsets after staining with an antibody to CXCR2 or isotype control.**D.** Isotype-corrected cell surface expression of CXCR2 on mouse monocyte subsets in *Apoe*^*−/−*^ mice fed normal chow. *Denotes significant increase in classical monocytes in mice on HFD.**E.** Serum mCXCL1 levels in *Apoe*^*−/−*^ mice fed normal chow or HFD for 8 weeks. *Denotes significant differences.**F.** Analysis of classical monocyte counts in blood, BM, and spleen 1 h after injection of PBS or recombinant murine CXCL1 (40 µg/kg). *Denotes significant differences.**G–I.** *Apoe*^*−/−*^ mice were fed a HFD for 4 weeks and injected (5 µg i.p., daily during first week, 3×/week in subsequent weeks) with isotype control or an antibody to mCXCL1. All data are expressed as mean ± SD. *Denotes significant difference between indicated groups. *n* = 8 for each group (Students *t*-test). (**G**) Absolute number of circulating classical monocytes in *Apoe*^*−/−*^ mice before and after 1 and 4 weeks of feeding HFD. (**H**) Lesion size in the aortic root. (**I**) Absolute numbers of classical monocytes (left) and macrophages (right) in the aorta, as assessed by flow cytometry.

Hypercholesterolemia has been reported to affect counts, phenotype, and function of peripheral blood leucocyte subsets. Increased monocyte counts in mice receiving HFD have been attributed to a selective expansion of the classical monocyte subset (Swirski et al, 2007). In our hands, *Apoe*^*−/−*^ mice fed a HFD exhibited a leucocytosis comprised of increased counts of neutrophils and classical monocytes (Fig 2A and B).

We next aimed at elucidating mechanisms underlying HFD-induced monocytosis. Prolonged life span, reduced conversion, and enhanced production have been implicated in causing HFD-mediated classical monocytosis (Murphy et al, 2011; Swirski et al, 2007). Hence, we examined facilitated mobilization as complementary mechanisms of classical monocytosis. The CCR2 ligands CCL2/MCP-1 and CCL7/MCP-3 are crucially involved in the mobilization of classical monocytes from the BM under steady-state and inflammatory conditions (Tsou et al, 2007). However, neither CCL2 nor CCL7 serum levels were found to be increased under HFD (Supporting Information Fig 5). Accordingly, *Ccr2*^*−/−*^*Apoe*^*−/−*^ mice displayed reduced circulating classical monocyte counts at steady-state but a significant increase after HFD for 8 weeks by a relative degree similar to that observed in *Ccr2*^*+/+*^*Apoe*^*−/−*^ mice (Supporting Information Fig 6), suggesting a mechanism independent of the CCR2 axis to be responsible for HFD-induced monocytosis. Likewise, comparable increases in classical monocyte counts were found in *Ccr1*^*−/−*^*Apoe*^*−/−*^*, Ccr5*^*−/−*^*Apoe*^*−/−*^, and *Cx*_*3*_*cr1*^*−/−*^*Apoe*^*−/−*^ mice (Supporting Information Fig 6), indicating a dispensable role of these receptors in HFD-induced classical monocytosis.

Since neutrophil homeostasis in steady-state and during hypercholesterolemia is regulated via the CXCR2-CXCL1 axis (Drechsler et al, 2010; Martin et al, 2003), we investigated whether this also applies to classical monocytes. We found CXCR2 to be expressed on both monocyte subsets with higher surface levels on classical monocytes (Fig 2C and D). Upon HFD, CXCR2 expression was increased on circulating classical monocytes but remained unaltered on non-classical monocytes (Supporting Information Fig 7). In addition, serum levels of mCXCL1 were significantly elevated in *Apoe*^*−/−*^mice fed a HFD (Fig 2E). Thus, both lines of evidence imply a potential involvement of CXCL1-CXCR2 in the regulation of classical monocytes homeostasis under HFD. The ability of CXCL1 to mobilize classical monocytes was further tested by intravenous injection of rmCXCL1 (Fig 2F). Whereas circulating non-classical monocyte levels remained stable, blood classical monocyte levels increased with concomitant decreases in BM and spleen (Fig 2F), the latter being a recently identified reservoir for monocytes (Swirski et al, 2009). To prove the importance of CXCL1 in HFD-induced monocytosis, we treated *Apoe*^*−/−*^ mice on HFD with an antibody to CXCL1 or isotype control. Whereas classical monocyte counts significantly increased in mice treated with isotype control, this was prevented by treatment with the anti-CXCL1 antibody (Fig 2G). In line, numbers of classical monocytes in spleen and BM showed a tendency towards an increase in mice receiving the anti-CXCL1 antibody (Supporting Information Fig 8). Aortic root sections of anti-CXCL1 treated mice displayed smaller lesions (Fig 2H) characterized by lower numbers of lesional classical monocytes and macrophages (Fig 2I). The relevance of CXCL1 in patients was further assessed in plasma of a previously described patient cohort with moderate hypercholesterolemia (Garlichs et al, 2001). In these patients, we found increased levels of CXCL1 as compared to controls (Supporting Information Fig 9). Taken together, the increase in CXCL1 levels in conjunction with a differential CXCR2 expression pattern indicates that this chemokine/receptor-axis is crucially involved in mediating the HFD-induced mobilization of classical monocytes.

### Arterial recruitment of classical monocytes depends on CCR1 and CCR5

**Figure 3:**
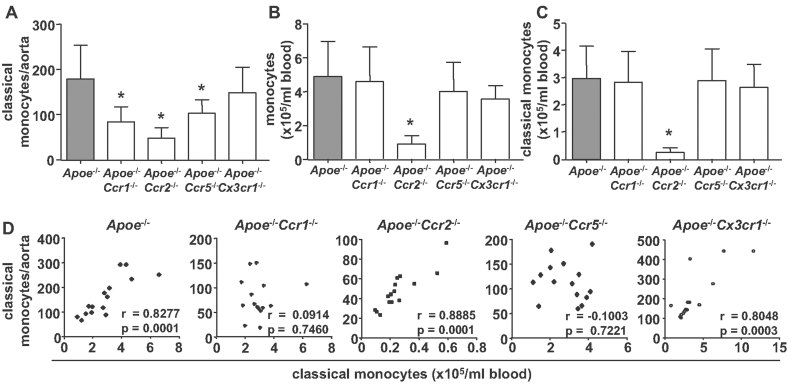
Correlating circulating and aortic monocyte counts reveals importance of CCR1 and CCR5 in recruitment of classical monocytes **A.** Absolute numbers of classical monocytes in aortas of indicated mouse strains after HFD for 8 weeks.**B,C.** Circulating total monocyte (**B**) and classical monocyte (**C**) counts in indicated mouse strains after 8 weeks of HFD, as analysed by flow cytometry. All data are expressed as mean ± SD. *Denotes significant differences between groups. *n* = 15 for each group (One-way ANOVA with Dunnett *post hoc* test).**D.** Correlation between aortic and circulating classical monocyte counts in indicated mouse strains after 8 weeks of HFD (Pearson correlation).

Previous studies have suggested pivotal contributions of CCR2 and CX_3_CR1 to monocyte and macrophage accumulation in atherosclerotic lesions (Combadiere et al, 2008; Tacke et al, 2007). However, leucocyte accumulation at sites of inflammation is regulated at various levels, namely mobilization from sites of production, recruitment, and life span. Since CCR2 is crucial for monocyte mobilization during inflammation (Serbina & Pamer, 2006), while CX_3_CR1 confers survival signals in monocytes and plaque macrophages (Landsman et al, 2009), we aimed at discerning such mechanisms from recruitment. Given the dominant role of classical monocytes in atheroprogression established herein, we evaluated the relevance of CCR1, CCR2, CCR5, and CX_3_CR1 in the recruitment of this subset to atherosclerotic arteries only. Notably, HFD did not alter surface expression of chemokine receptors on classical monocytes, although mRNA levels were up-regulated (Supporting Information Fig 7). In addition, expression of decoy receptors D6 and CXCR7 on the surface of classical monocytes was not affected by HFD (Supporting Information Fig 10). Similarly, expression of CD44, which can serve as CCL5 co-receptor, did not change following HFD (Supporting Information Fig 10). Next, we quantified classical monocyte counts in aortic cell suspensions of *Apoe*^*−/−*^*, Apoe*^*−/−*^*Ccr1*^*−/−*^*, Apoe*^*−/−*^*Ccr2*^*−/−*^*, Apoe*^*−/−*^*Ccr5*^*−/−*^, and *Apoe*^*−/−*^*Cx*_*3*_*cr1*^*−/−*^ mice by flow cytometry (Supporting Information Fig 11). Compared to control *Apoe*^*−/−*^ mice, the number of classical monocytes was significantly reduced in aortas of atherosclerotic mice lacking CCR1, CCR2, and CCR5 but not of those lacking CX_3_CR1 both after 4 weeks (Supporting Information Table 4) and 8 weeks (Fig 3A) of HFD. In line with a role in mobilization (Serbina & Pamer, 2006; Tsou et al, 2007), *Apoe*^*−/−*^*Ccr2*^*−/−*^ mice displayed reduced classical monocyte counts in the circulation, whereas no differences were observed in *Apoe*^*−/−*^*Ccr1*^*−/−*^*, Apoe*^*−/−*^*Ccr5*^*−/−*^, and *Apoe*^*−/−*^*Cx*_*3*_*cr1*^*−/−*^ mice (Fig 3B and C). To further discriminate between homeostasis and recruitment, we correlated the counts of circulating and aortic classical monocytes. While the two parameters were strongly correlated in *Apoe*^*−/−*^, *Apoe*^*−/−*^*Ccr2*^*−/−*^, and *Apoe*^*−/−*^*Cx*_*3*_*cr1*^*−/−*^ mice, no correlation was observed in *Apoe*^*−/−*^*Ccr1*^*−/−*^ or *Apoe*^*−/−*^*Ccr5*^*−/−*^ mice (Fig 3D), suggesting a recruitment deficit in the latter two strains. Whereas macrophage accumulation was more markedly reduced in *Apoe*^*−/−*^*Cx*_*3*_*cr1*^*−/−*^ mice than in *Apoe*^*−/−*^*Ccr2*^*−/−*^ and *Apoe*^*−/−*^*Ccr5*^*−/−*^ mice at both time points, consistent with a role of CX_3_CR1 monocytes and in macrophage survival (Landsman et al, 2009), the absence of CCR1 limited macrophage accumulation at early time points but appeared to favor macrophage accumulation at later stages (Supporting Information Table 4).

**Figure 4:**
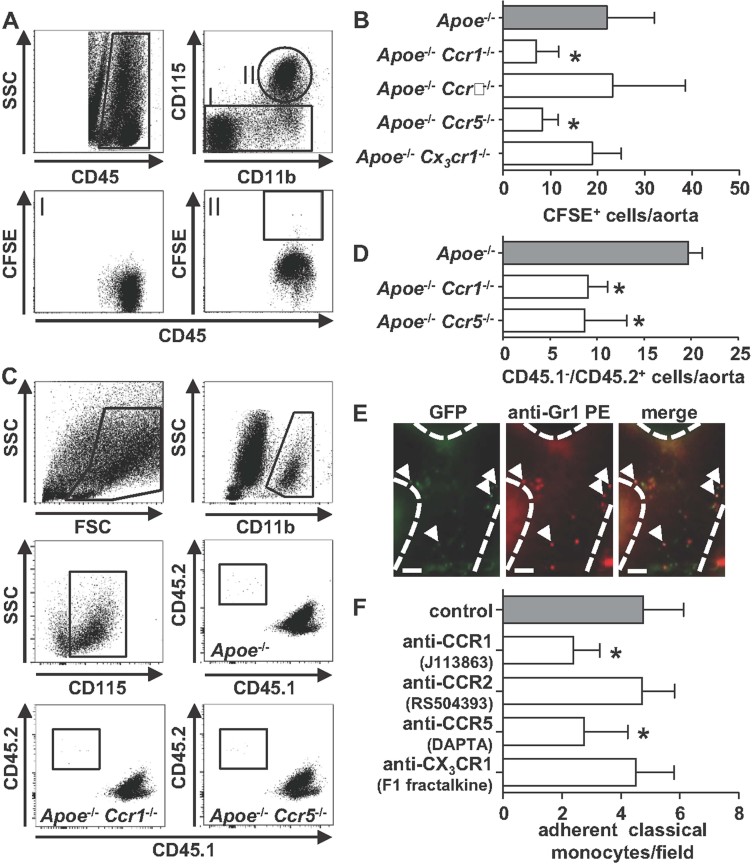
CCR1 and CCR5 mediate arterial classical monocyte infiltration **A,B.** Classical monocytes (10^6^) of indicated donor mouse strains were injected into *Apoe*^*−/−*^ recipients after labelling with the cell tracker CFSE and allowed to circulate for 24 h. Both donor mice and recipients had been on HFD for 8 weeks. Gating strategy (**A**) and absolute numbers of labeled cells in the aorta quantified by flow cytometry (**B**) are depicted. *Denotes significant differences compared to injection of classical monocytes from *Apoe*^*−/−*^ donor mice. *n* = 7 for each group (Kruskal–Wallis with Dunns *post hoc* test).**C,D.** Classical CD45.2^+^ monocytes (10^6^) of indicated donor mouse strains were injected into CD45.1/*Ldlr*^*−/−*^ recipients and allowed to circulate for 24 h. Both donor mice and recipients had been on HFD for 8 weeks. Gating strategy (**C**) and absolute numbers of CD45.2^+^ monocytes in the aorta as quantified by flow cytometry (**D**) are depicted. *Denotes significant differences compared to injection of classical monocytes from *Apoe*^*−/−*^ donor mice. *n* = 7 for each group (Kruskal–Wallis with Dunns *post hoc* test).**E.** Visualization of leucocyte adhesion to the carotid artery of *Apoe*^*−/−*^*Cx3cr1*^*epgf/+*^ mice having been on HFD for 8 weeks. To discriminate between classical and non-classical monocytes a PE-conjugated antibody to Gr1 was injected. Scale bar = 50 µm.**F.** Quantification of adhesion of classical monocytes to carotid arteries of *Apoe*^*−/−*^*Cx3cr1*^*epgf/+*^ mice having been on HFD for 8 weeks and having received a single dose of indicated chemokine receptor antagonist 1 h prior to experimentation. All data are expressed as mean ± SD. *Denotes significant differences compared to control mice. *n* = 7–8 (Kruskal–Wallis with Dunns *post hoc* test) for each group.

To corroborate these findings, we performed adoptive transfer experiments using classical monocytes sorted from BM of *Apoe*^*−/−*^*, Apoe*^*−/−*^*Ccr1*^*−/−*^*, Apoe*^*−/−*^*Ccr2*^*−/−*^*, Apoe*^*−/−*^*Ccr5*^*−/−*^, and *Apoe*^*−/−*^*Cx*_*3*_*cr1*^*−/−*^ mice. From each donor strain, 10^6^ classical monocytes were labelled with carboxyfluorescein succinimidyl ester (CFSE) as a cell tracker and injected intravenously into *Apoe*^*−/−*^ mice that had been on HFD for 8 weeks. After 24 h, adoptively transferred classical monocytes were quantified in aortic homogenates of recipient mice (Fig 4A and B and Supporting Information Fig 12). The recruitment of classical monocytes deficient in CCR1 or CCR5 but not of those deficient in CCR2 or CX_3_CR1 was severely diminished, when compared to monocytes with intact chemokine receptor profile. To further substantiate these findings, we employed the same strategy but instead used the CD45.1/CD45.2 system to monitor aortic recruitment of classical monocytes. Based on improved discrimination of donor cells within the aortas of CD45.1/*Ldlr*^*−/−*^ recipient mice, the results corroborate the importance of CCR1 and CCR5 for arterial monocyte influx (Fig 4C and D).

This approach was further complemented by intravital microscopy of the carotid artery using *Apoe*^*−/−*^*Cx*_*3*_*cr1*^*egfp/+*^ reporter mice. To specifically track classical monocytes, a PE-conjugated antibody to Gr1 was injected, rendering classical monocytes red/green fluorescent (Fig 4E). The involvement of CCR1, CCR2, CCR5, or CX_3_CR1 in the adhesion of classical monocytes to carotid arteries of *Apoe*^*−/−*^*Cx*_*3*_*cr1*^*egfp/+*^ mice after 8 weeks of HFD was investigated by intraperitoneal administration of specific antagonists to the respective chemokine receptor 1 h prior to recording. Inhibition of CCR1 or CCR5 markedly reduced luminal adhesion of classical monocytes, an effect not observed by the presence of antagonists to CCR2 or CX_3_CR1 (Fig 4F). Collectively, these data point at a prevalent function of CCR1 and CCR5 in the recruitment of classical monocytes under acute as well as chronic inflammatory and atherogenic conditions.

## DISCUSSION

The importance of monocytes/macrophages in atherosclerosis is widely acknowledged. However, the principal mechanisms of their proatherogenic function, namely differential contributions of monocyte subsets, the control of their homeostasis and recruitment in hypercholesterolemia and atherosclerosis remain insufficiently defined. Herein, we have unequivocally established a predominant role of classical monocytes in atheroprogression. As a major risk factor for atherosclerosis, we found HFD-induced hypercholesterolemia to enhance classical monocytes counts by engaging the mCXCL1-CXCR2 axis. Finally and contrary to previous reports, the CCL5 receptors CCR1 and CCR5 were identified to be essential for the recruitment of classical monocytes to atherosclerotic arteries.

Our data provide several lines of evidence for differential roles of the chemokine receptors CXCR2, CCR1, CCR2, CCR5, and CX_3_CR1 in arterial monocyte accumulation. Previous studies revealed diminished atherosclerotic plaque formation in mice deficient in these receptors or their respective ligands (Gautier et al, 2009). However, chemokine receptors do not only control monocyte recruitment at sites of inflammation but also their mobilization from the BM, and their life span (Gautier et al, 2009). CXCR2 has been shown to mediate recruitment of monocytes and neutrophils to atherosclerotic arteries (Boisvert et al, 1998; Drechsler et al, 2010), in part explaining the reduced atherosclerotic lesion formation in CXCR2-deficient mice. An alternative role of CXCR2 in atherogenesis was unveiled by our findings that its ligand CXCL1 mediates mobilization of classical monocytes under hypercholesterolemia. Under these conditions, CXCL1 may be derived from activated endothelium covering atherosclerotic lesions (Zhou et al, 2011). The importance of CXCL1 in atherosclerotic lesion formation and macrophage accumulation was established by CXCL1 neutralization (Boisvert et al, 2006), which may be explained by the influence on monocyte mobilization under HFD identified herein. Elevated mCXCL1 levels under HFD not only impart neutrophilia but also promote mobilization of classical monocytes exhibiting higher CXCR2 surface expression than their non-classical counterparts. Mobilization of classical monocytes along with higher circulating monocyte counts correlate with plaque sizes (Combadiere et al, 2008; Swirski et al, 2007). In conjunction with its role in arterial cell recruitment, the atherogenic effects of CXCR2 may thus be attributable to HFD-mediated effects on monocyte homeostasis.

Whereas various studies in *Ccr2*^*−/−*^ mice support an important role of CCR2 in monocyte extravasation, three different approaches employed in this study to discern its effects on homeostasis and recruitment, clearly imply that arterial recruitment of classical monocytes does not require CCR2. Accordingly, several recent studies using adoptive transfer strategies suggest that CCR2 is dispensable for peripheral recruitment of classical monocytes (Engel et al, 2008; Serbina & Pamer, 2006). Hence, reduced atherosclerotic lesion sizes in *Ccr2*^*−/−*^*Apoe*^−/−^ mice (Boring et al, 1998) may primarily result from lower counts of circulating classical monoctyes rather than defects in their recruitment.

In contrast to CCR2 and CX_3_CR1, CCR1 and CCR5 were found to be crucially involved in the recruitment of classical monocytes to atherosclerotic arteries. Both receptors share an overlapping spectrum of ligands among them CCL3 and CCL5, which are present in atherosclerotic lesions through expression or deposition. While global blockade of CCL5 receptors using Met-CCL5 (Veillard et al, 2004) and CCR5 deficiency are associated with reduced atherosclerotic lesion size, CCR1-deficiency (somatic or in BM) was shown to exacerbate plaque formation (Braunersreuther et al, 2007). Notably, the effects of plaque development in *Ccr1*^*−/−*^ mice appear to be highly stage-dependent. When compared to control mice, atherosclerotic lesions in *Ccr1*^*−/−*^*Apoe*^−/−^ mice are smaller after 1 month of HFD, comparable at 2 months and larger at 3 months of HFD. Whereas early-stage effects may be due to decreased recruitment of neutrophils (Drechsler et al, 2010) or monocytes, the exacerbation at later stages may reflect effects favoring macrophage accumulation or a stimulation of T-cell-driven immune responses (Braunersreuther et al, 2007). In contrast, findings for CCR5 are much clearer, *i.e.* mice deficient in CCR5 exhibit smaller lesions with reduced numbers of mononuclear cells in several models (Braunersreuther et al, 2007). The non-redundant importance of both CCR1 and CCR5 identified herein can be explained by a concept proposing a division of labour during the emigration process, where CCR1 mediates monocyte arrest, CCR5 supports monocyte spreading and both contribute to transendothelial migration towards CCL5 (Weber et al, 2001).

With the emergence of two monocyte subsets, a multistep model would envision that the relative surface expression of adhesion molecules and chemokine receptors determines the recruitment behaviour of each subset. Hence, higher CCR1 levels on classical monocytes may reflect its relevance for the recruitment of this monocyte subset, whereas CCR5 is equally expressed on both subsets, thus partially explaining an involvement in the rare recruitment of non-classical monocytes (Tacke et al, 2007). The prominent role of CCR1 and CCR5 can also be related to the deposition of platelet-derived chemokines mediating proatherogenic monocyte adhesion on endothelium (von Hundelshausen et al, 2001). Namely, the CCR1 and CCR5 agonist CCL5 triggers monocyte arrest, an effect further enhanced when CCL5 interacts with CXCL4 (Koenen et al, 2009). The *in vivo* relevance of this synergistic interaction was substantiated by findings that disruption of CCL5-CXCL4 heteromer formation markedly inhibited atherosclerotic lesion formation (Koenen et al, 2009). In addition, platelet-derived CCL5 can promote arterial recruitment of neutrophils via engagement of CCR1 and CCR5 (Drechsler et al, 2010). By release and deposition of granule contents, neutrophils specifically induce adhesion and recruitment of classical monocytes (Doring et al, 2012; Soehnlein et al, 2008; Wantha et al, 2013). Hence, the prominent role of CCR1 and CCR5 may in part reflect the contribution of platelet- and neutrophil-borne proteins with recruitment activity.

While previous data have suggested a function of CX_3_CR1 in the recruitment of classical monocytes (Tacke et al, 2007) despite lower expression than in non-classical monocytes, recent data offer an alternative explanation (Landsman et al, 2009). Beyond recruitment, the CX_3_CL1-CX_3_CR1 axis confers essential survival signals for monocytes, whereas its absence leads to increased death of plaque monocytes and foam cells, providing a mechanism for reduced plaque sizes of CX_3_CR1-deficient mice (Landsman et al, 2009; Lesnik et al, 2003). Accordingly, our data indicate that CX_3_CR1 is dispensable or redundant in the arterial recruitment of classical monocytes but that its role in arterial recruitment of non-classical monocytes merits further investigation.

A group of silent or decoy receptors able to sequester chemokines (Mantovani et al, 2006) may also be important for monocyte trafficking in atherosclerosis. D6, an important member of the decoy receptor family, binds a broad range of CCR1, CCR2, and CCR5 ligands, prevents excessive infiltration of classical monocytes and neutrophils into the myocardium in a mouse model of myocardial infarction (Cochain et al, 2012). In addition, CXCR7 acts as a scavenger for CXCL12, a chemokine important in the retention of stem cells and neutrophils in the BM (Naumann et al, 2010; Zernecke et al, 2008). The relevance of chemokine-scavenging receptors to atherogenic mobilization and recruitment of myeloid cell subsets remains to be investigated. Arrest chemokines, such as CXCL2, CXCL8, and CCL5 (alone or as heterodimer with CXCL4) bind to the glycosaminoglycans on the surface of endothelial cells, immobilize, and mediate firm adhesion of rolling monocytes. The association of chemokines with heparan sulphate can immobilize chemokines on the vessel wall to provide strong and localized signals for integrin activation (Ley, 2003). The interaction of chemokines with glycosaminoglycans or heparan sulphate-decorated CD44 may strengthen chemokine function by various mechanisms, including induction of conformational changes with enhanced activity, protection from proteolytic inactivation, and induction of dimer or heteromer formation (Rot, 2010). Notably, the expression of D6, CXCR7, and CD44 shaping chemokine function was unaltered by HFD in our model.

In conclusion, our data establish the quintessential impact of classical monocytes on atheroprogression. Our findings further identify sequential contributions of the CXCL1/CXCR2 axis in the proatherogenic mobilization of classical monocytes and of the CCL5 receptors CCR1 and CCR5 in the control of their recruitment to atherosclerotic arteries. In addition, further experimentation is needed to investigate to what extend chemokines control post-recruitment processes contributing to lesional macrophage accumulation.

## MATERIALS AND METHODS

### Animals

Male *Apoe*^*−/−*^ mice and chemokine receptor-deficient male *Apoe*^*−/−*^ mice have been previously described (Braunersreuther et al, 2007; Landsman et al, 2009; Schober et al, 2004). All strains were backcrossed for at least 10 generations to the C57Bl/6 background. Mice received HFD (21% fat, 0.15% cholesterol, Altromin) for indicated time points resulting in similar lipid levels in all strains (data not shown).

### *In vivo* experiments

To assess the role of monocyte subsets in atheroprogression, 6 weeks-old *Apoe*^*−/−*^mice were fed a HFD for a total of 8 weeks. After 4 weeks of HFD, stable leucopenia was induced by repeated CPM injection (100 mg/kg BW, 2×/week). To reconstitute leucocytes, age- and sex-matched *Apoe*^*−/−*^ mice receiving HFD for an equal time period were exsanguinated. Blood was labelled with antibodies to CD45, Gr1, and CD115, and individual monocyte subsets were depleted by FACS sorting. Donor leucocytes were injected i.v. 2×/week using one donor mouse per recipient each 1 day after CPM application.

For adoptive transfer studies, classical monocytes were isolated from BM by FACS sorting using antibodies to CD45, CD115, and Gr1. After labelling the monocytes with CFSE, 10^6^ cells were adoptively transferred by tail vein injection to *Apoe*^*−/−*^ mice. Twenty-four hours after transfer, aortas and hearts of recipient mice were collected for further analysis. For monocyte mobilization studies, rmCXCL1/KC (Peprotech) was injected i.v. at 40 µg/kg. After 1 h, blood was drawn and mice were sacrificed to harvest bones and spleens. Serum CXCL1 in mice was neutralized by daily administration of 5 µg of anti-CXCL1 antibody (clone 124014) or IgG isotype control (clone 54447, both R&D Systems) for 1 week and for 3 consecutive weeks every other day.

**The paper explained**

PROBLEM:

Monocytes and macrophages are the most abundant white blood cell subsets in atherosclerotic lesions. To enter atherosclerotic plaques, monocytes employ specific guide cues called chemokines, which induce monocyte migration via binding to chemokine receptors. However, these ligands and receptors may not just be important in emigration of monocytes from the blood to the arteries, but they may also control mobilization from the bone marrow (BM) or the life span of monocytes. Hence, the aim of this study was to investigate the importance of various chemokines and their receptors in atherogenic monocyte mobilization and recruitment.

RESULTS:

With the existence of two murine monocyte subsets we first wanted to investigate which of these two subsets is more important in early stages of atherosclerosis. Based on a depletion and reconstitution strategy, we could identify an important role for classical monocytes. Under conditions of hypercholesterolemia, a classical risk factor for atherosclerosis, classical monocytes are produced in larger numbers in the BM and spleen and based on data from this study we identify that the CXCL1-CXCR2 axis is important in mobilization of classical monocytes from these sites of production. In contrast to this and to previously published data, CCR1 and CCR5 but not CCR2 or CX3CR1 were found to be important chemokine receptors mediating the accumulation of classical monocytes in atherosclerotic lesions.

IMPACT:

Collectively, these data establish the impact of classical monocytes on atheroprogression, and identify a sequential role of CXCL1 in atherogenic mobilization of classical monocytes and CCR1/CCR5 in arterial recruitment of classical monocytes. Hence, we here identify three potential targets for therapeutic intervention and further studies are warranted to dissect the specificity of such interventional strategies.

### Flow cytometry

Staining of single cell suspensions of blood, BM, spleen, or aorta was conducted using combinations of antibodies specific for CCR1-purified (Imgenex, IMG329), CCR2-purified (Epitomics, E68), CXCR2-purified (R&D Systems, clone 242216), CXCR7 (BioLegend, 8F11-M16), CX_3_CR1 (R&D Systems, AF5825), CCR5-biotinylated (BD, C34-3448), CD115-PE (eBioscience, AFS98), CD11b-PerCp/PE-Cy7 (BD, M1/70), CD44-PerCp (eBioscience, IM7), CD45-APC-Cy (BD, 30-F11), CD45.1-PE-Cy7 (eBioscience, A20), CD45.2-APC (eBioscience, 104), D6-purified (ThermoScientific), F4/80-APC (eBioscience, BM8), CD62L (eBioscience, MEL-14), Gr1-APC/PerCP (eBioscience/BD, RB6-8C5), anti-ratIgG-FITC (eBioscience, 11-4811-85), anti-rabbit IgG-FITC (Sigma–Aldrich), SAV-PE-Cy7 (BD). Before cell staining, red blood cell lysis was performed using appropriate volume of lysis buffer (150 mM NH_4_Cl; 10 mM KHCO_3_; 0.1 mM diNaEDTA, pH 7.4). Cells were washed with HBSS and directly analysed by flow cytometry using a FACSCantoII (BD). Absolute cell numbers were assessed by use of CountBright™ absolute counting beads (Invitrogen). Data were analysed with FlowJo Software (Tree Star Inc.). To assess expression levels of interest, geometrical mean fluorescence intensity (MFI) after subtracting the fluorescence minus one (FMO) control was calculated.

### Histology, immunohistochemistry, and immunofluorescence

The extent of atherosclerosis was assessed in aortic root sections by oil-red-O staining (Sigma–Aldrich), followed by computerized image analysis and quantification (Leica Qwin Imaging software). Collagen content was evaluated after Sirius red staining. To define monocyte/macrophage numbers in atherosclerotic plaque area, frozen sections of aortic roots were washed with PBS for at least 5 min followed by an overnight incubation with a 1:400 dilution of anti Mac-2 antibody at 4°C. After incubation with secondary Cy-3 conjugated antibody for 30 min at room temperature, sections were analysed. To assess the accumulation of CD45.2 donor cells within aortic root sections of CPM treated CD45.1 mice, slides were treated with target retrieval solution (Dako). After blocking, sections were stained for CD45.1 over 60 min at RT with an anti-CD45.1 primary (Abcam, A20) and an anti-mouse FITC-conjugated secondary (Jackson ImmunoResearch) antibody. After a second treatment with blocking solution containing mouse serum and avidin sections were subsequently stained with a biotinylated anti-CD45.2 primary (eBioscience, 104) antibody and streptavidin-DyLight549 (Vector Laboratories). All sections were analysed using a Leica DMLB fluorescence microscope and charge couple device (CCD) camera. Furthermore, TUNEL staining was performed using *In Situ* Cell Death Detection Kit, TMR red (Roche) to assess the number of apoptotic/necrotic cells within aortic root sections.

### Construction of the CX_3_CR1 Antagonist

An expression construct for the CX_3_CR1-antagonist F1-fractalkine (Dorgham et al, 2009) was ordered at Genscript (Piscataway) as *E. coli* codon-optimized cDNA cloned in pET26b (Merck). Recombinant F1-fractalkine containing an N-terminal cleavable His-tag (MHHHHHHWVDDDDK–^1^ILDN… TRNGG^92^) was expressed in BL21(DE3)Star cells (Invitrogen) cultured in LB medium using a fermenter (Lambda Instruments) for 4 h at 37°C. Insoluble inclusion bodies were washed and isolated by repeated centrifugation, solubilized in Guanidine-HCl and refolded by rapid dilution in native buffer essentially as described (Mizoue et al, 1999). The refolded His-F1-fractalkine was enriched by sequential Ni-NTA and cation-exchange chromatography. Finally, the leader peptide was removed by overnight digestion with recombinant enterokinase (Merck) (1 U/mg protein) and active F1-fractalkine was separated from uncleaved His-F1-fractalkine after cation exchange chromatography. F1-fractalkine was dialysed in 0.1% TFA and stored lyophilized at −30°C.

### Intravital microscopy

Intravital microscopy of the carotid artery was performed in *Cx*_*3*_*cr1*^*egfp/+*^*Apoe*^*−/−*^ mice as described (Drechsler et al, 2010). J113863 to CCR1 (5 mg/kg), RS504393 to CCR2 (5 mg/kg), DAPTA to CCR5 (1 mg/kg), or the above-designed antagonist F1-fractalkine to CX_3_CR1 (5 mg/kg) were injected i.p. 1 h prior to recording. A PE-conjugated antibody to Gr1 (5 µg) was instilled via a jugular vein catheter 15 min prior to recording. Intravital microscopy was performed using an Olympus BX51 microscope equipped with a beam splitter to enable synchronized dual-channel recording, a Hamamatsu 9100-02 EMCCD camera, and a 10× saline-immersion objective. For image acquisition and analysis Olympus cell^r^ software was used.

### Lipid detection

Serum levels of cholesterol or triglycerides were assessed by EnzyChrom™ Assay Kits (BioAssay Systems).

### CXCL1 measurements in human plasma

Human plasma samples from patients with moderate hypercholesterolemia and respective controls (Garlichs et al, 2001) were analysed for CXCL1 by use of a commercially available ELISA kit (Quantikine, R&D systems).

### ELISA

Different ready-to-use ELISA systems were employed according to manufacturer instructions. Murine MCP-1 and CXCL1 were determined using Quantikine ELISAs (R&D Systems). Serum levels of MCP-3 were detected by Instant ELISA (eBioscience).

### PCR Array

Employing RT^2^ Profiler PCR Array (SABiosciences) expression of genes encoding chemokines and their receptors could be investigated. RNA of cells obtained from FACS was isolated using RNeasy Micro Kit (Qiagen) and quantified by measuring the absorbance at 260 nm (A260) in a spectrophotometer. Using the RT^2^ First Strand Kit (SABiosciences) cDNA has been generated and checked for quality and efficiency of reverse transcription by RT^2^RNA QC PCR Array. In case the quality of cDNA met the demands, RT^2^ Profiler PCR Array for mouse chemokines and receptors was performed.

### Statistics

All continuous data are expressed as mean ± SD. Statistical calculations were performed using GraphPad Prism 5 (GraphPad Software Inc.). After calculating for normality by D'Agostino Pearson omnibus test either unpaired Student's *t*-test, One-way ANOVA with Newman–Keuls multiple comparison or nonparametric tests such as Mann–Whitney test, or Kruskal–Wallis test with *post hoc* Dunn test were used. *Indicates a *p*-value < 0.05.

## Supplementary material

As a service to our authors and readers, this journal provides supporting information supplied by the authors. Such materials are peer reviewed and may be re-organized for online delivery, but are not copy-edited or typeset. Technical support issues arising from supporting information (other than missing files) should be addressed to the authors.
