# Supplementary material for: Distinct functions of chemokine receptor axes in the atherogenic mobilization and recruitment of classical monocytes
Source: EMBO Mol Med. 2013 Feb 18;5(3):471–81. doi: 10.1002/emmm.201201717 (PMC3598085; doi:10.1002/emmm.201201717)
Supplement: Supplementary file 1 [file emmm0005-0471-SD1.pdf]

EMBO Molecular Medicine

Distinct function of chemokine receptor axes in the atherogenic mobilization and recruitment of classical monocytes

Oliver Soehnlein, Maik Drechsler, Yvonne Döring, Dirk Lievens, Helene Hartwig, Klaus Kemmerich, Almudena Ortega-Gómez, Manuela Mandl, Santosh Vijayan, Delia Projahn, Christoph D. Garlichs, Rory R. Koenen, Mihail Hristov, Esther Lutgens, Alma Zernecke, Christian Weber

Corresponding author: Oliver Soehnlein, Ludwig-Maximilians University Munich

Review timeline:

Submission date:	09 July 2012
Editorial Decision:	24 August 2012
Revision received:	13 November 2012
Editorial Decision:	17 December 2012
Revision received:	19 December 2012
Accepted:	20 December 2012

Transaction Report:

(Note: With the exception of the correction of typographical or spelling errors that could be a source of ambiguity, letters and reports are not edited. The original formatting of letters and referee reports may not be reflected in this compilation.)

Editors: Anneke Funk / Roberto Buccione

2nd Editorial Decision 24 August 2012

Thank you for the submission of your manuscript "Distinct function of chemokine receptor axes in the atherogenic mobilization and recruitment of classical monocytes" to EMBO Molecular Medicine and please accept my apologies for the delayed reply. We have now finally heard back from the three referees whom we asked to evaluate your manuscript. You will see that they find the topic of your manuscript potentially interesting. However, they also raise significant concerns on the study, which should be addressed in a major revision of the manuscript.

In particular, reviewer #2 highlights that the data regarding specific involvement of CCR1 and CCR5 in monocyte accumulation should be strengthened. Importantly, reviewer #1 feels that potential confounding effects of the positive selection should be excluded.

Given the balance of these evaluations, we feel that we can consider a revision of your manuscript if you can convincingly address the issues that have been raised within the time constraints outlined below.

Revised manuscripts should be submitted within three months of a request for revision. They will otherwise be treated as new submissions, unless arranged otherwise with the editor.

I look forward to seeing a revised form of your manuscript as soon as possible.

***** Reviewer's comments *****

Referee #1:

This paper from the Soehnlein and colleagues probes some of the mechanistic consequences of the observation that classically activated monocytes levels increase in hypercholesterolemic mice.

By a positive-selection, adoptive transfer approach in animals that have been made leukopenic by cyclophosphamide administration, they showed that classically activated monocytes aggravate early atherogenesis. In an extensive series of experiments, the authors explore the contribution of chemokines and their receptors in this phenomenon. Their data support a role of CCR 1 and CCR 5, and conversely do not find that CCR2 or CX3CR1 contribute to monocyte recruitment. Aspects of this work are novel, and the mechanistic insights add value to the field of atherogensis in mice.

Cells for reconstitution were prepared by positive selection. Could this treatment have had confounding effects by activating WBC or altering their fate upon transfer?

Does the cyclophosphamide treatment affect lymphocyte functions related to atherogenesis?

Does cyclophosphamide have a general effect of collagen? All cyclophosphamide treated animals had lower levels of collage regardless of the monocyte population reconstituted (Fig 1G).

Why is apoptosis greater in the CM depleted lesions (fig 1F)?

With respect to the experiments in fig 3, only one time point is reported in the effects of chemokine knockouts on CM amounts is lesions. How long do CM persist in lesions before maturation to macrophages, and could the genetic manipulations alter this rate, such that a kinetic analysis would be more informative than a single timepoint?

The authors are quite aware of what they call "stage-dependent" effects of interventions on atherosclerosis. In view of this important issue, they should try to temper throughout this manuscript the strength of their conclusions that are based on study of only one time point.

The authors should omit the speculation regarding targeting of HDL on page 11. This is unjustified given the current state of knowledge.

The authors should use a better term than "atherosclerotic endothelium" on page 10.)

The use of non-standard abbreviations is not helpful to the reader. The field is already confused by use of Gr and Ly6 nomenclature. Is the use of CM and NCM here needed?

Referee #2:

In the manuscript "Distinct function of chemokine receptor axes in the atherogenic mobilization and recruitment of classical monocytes" Soehnlein, Weber and colleagues reexamine the role of classical monocytes in atherosclerosis with an emphasis on chemokine receptors. They conclude that: (1) Ly-6C high classical monocytes are selectively atherogenic; (2) the CXCR2-CXCL1 chemokine axis is responsible for the mobilization of classical monocytes to the blood; (3) CCR1 and CCR5 are the main receptors that promote classical monocyte accumulation in lesions. Overall, the study recapitulates previous observations with new methods while challenging other findings. The question is whether the authors provided sufficient evidence to support their conclusions.

1. The first conclusion is that classical monocytes are atherogenic. This is the basis of Figure 1. The conclusion recapitulates previous studies with a new, tour-de-force technique. In fact, it's remarkable that the authors see such effects on disease simply by adoptively transferring monocytes. However, it is unclear from Figure 1 whether the effect has anything to do with the accumulation of monocytes. The authors show in Supporting Figure 8 that adoptively transfered monocytes are

indeed found in recipient blood. Do you also see adoptively transferred monocytes and macrophages in aorta? Given the data in Figure 1, lesional macrophages in group II should be CD45.2 and not CD45.1.

2. Next, the authors conclude on the basis of Figure 2 that CXCR2-CXCL1 axis is crucial to mobilize monocytes during HFD. The authors argue in Supplemental Figures 2 and 3 that the other chemokines typically associated with monocyte recruitment in atheorosclerosis are dispensable to hypercholesterolemia-induced atherosclerosis. The data are interesting. Is there statistical significance in G between HFD-isotype and HFD-anti-CXCL1? There should be if we are to conclude that CXCL1 is important. The increase shown by flow cytometry of CXCR2 on classical monocytes is modest. The authors should substantiate the finding with another method. Also, is there any impact on atherosclerosis with repeated anti-CXCL1?

3. Finally, the authors show that CCR1 and CCR5 but not CCR2 or CX3CR1 are involved in monocyte accumulation to lesions. This is the most controversial part of the paper and probably the most important. It must therefore be very convincing - unfortunately, it is not. First, the authors should report lesion size and number of macrophages in CCR1 and CCR5 apoE mice. In figure 3 the authors show reduced numbers of monocytes in the aorta in CCR1 and CCR5 mice which suggests a problem with influx but could also mean maturation, survival, exit. Second, the CFSE experiment is not convincing. There are almost no CFSE cells accumulating in lesions so even with the stars that denote statistical significance in Figure 4B the data are weak. The authors should perform experiments to more convincingly show that CCR1 and CCR5 are the essential chemokine receptors.

Overall, this is an interesting paper but has not yet convincingly proven its most important conclusions.

Referee #3:

Soehnlein et al describe in their study a new and very topical way to functionally differentiate between contributions of different monocyte subsets to atheroprogression. The authors have also extensively investigated the involvement of various chemokines and chemokine receptors and their conclusions about the underlying chemokine network seem sound and valid. This reviewer is, however, missing a treatment of non-signalling chemokine co-receptors in this otherwise highly interesting study. Before acceptance for publication it is therefore suggested to
- include references to decoy receptors such as D6 and discussions thereof
- include references to glycosaminoglycan(GAG) and proteoglycan chemokine co-receptors such as heparin sulfate and discussions thereof
- investigate (e.g. by real time PCR or by FACS analyses) the involvement of the above-mentioned non-signalling chemokine co-receptors
Since many chemokine-targeting therapies have failed for various reasons in the past, it is required to aim at a fairly complete picture of chemokines/receptor/co-receptor networks before seriously speculating about therapeutic targeting of CCL5/CCR1/CCR5 interactions.

1st Revision - authors' response 13 November 2012

Referee #1:

Cells for reconstitution were prepared by positive selection. Could this treatment have had confounding effects by activating WBC or altering their fate upon transfer?
Reply:
This question raised by the referee is certainly an important point that needs to be discussed. However, we would like to point out that groups II through IV received white blood cells exposed to the same cocktail of antibodies. Since FACS-sorting depletion of individual monocyte subsets had distinct effects on lesion sizes, we were confident that our antibody-based selection strategy did not

alter activation of white blood cells. To further investigate if the positive selection had an effect on leukocyte activation, we FACS-sorted white blood cells based on their FSC/SSC properties. In one instance the cells were incubated with the antibody cocktail used in figure 1 (anti-CD45, anti-CD115, anti-Gr1), whereas in the other instance they remained untouched. To assess leukocyte activation, we measured surface markers (CD11b, CD62L), the production of reactive oxygen species, and the exposure of phosphatidyl serine. In none of these measurements, antibody-based selection had a significant impact on function or phenotype of monocytes or neutrophils (new Supporting Information Figure 1).

Does the cyclophosphamide treatment affect lymphocyte functions related to atherogenesis?
Reply:
This is certainly an important question and our reply to this has various complex aspects. As for the question above, we would like to point out that groups I through IV received the same dose of cyclophosphamide (CPM). Hence, whatever the effect of CPM on lymphocyte or resident cell function may be, it can be considered to be the same in each of the groups. Beyond this, groups II through IV are repopulated with white blood cells (the majority of which are lymphocytes that can also be detected in the circulation, see Supporting Information Figure 2) which were not exposed to CPM and hence are functionally not impaired. Moreover, lesion sizes in groups I through IV are specifically modulated by presence or absence of classical monocytes making a major contribution of lymphocytes unlikely. Finally, in models of diet-induced atherosclerosis in *Apoe*$^{-/-}$ mice myeloid cells have a dominant role, while lymphocytes are known to play only minor roles (*e.g.* Dansky *et al.,* PNAS, 1997).

To experimentally assess the impact of CPM on lymphocyte function we repeated groups 0 and I with a smaller number of *Apoe*$^{-/-}$ mice. The capacity of lymphocytes to proliferate was tested with a

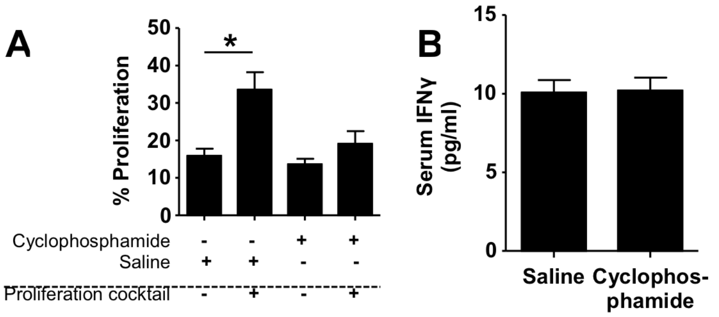

Cyclophosphamide affects lymphocyte proliferation but not IFNγ production. *Apoe*$^{-/-}$ mice received HFD for 8 weeks. During the last 4 weeks, mice were treated with cyclophosphamide (100 mg/kg BW, 2x/week, i.p.) or saline. **A:** Lymphocyte proliferation was assessed following stimulation with anti-CD3, anti-CD28, and IL2 (proliferation cocktail). **B:** IFNγ concentration in the serum as determined by ELISA.

cocktail containing anti-CD3, anti-CD28, and IL2 (see figure A above). In these experiments lymphocytes from CPM-treated mice proliferated, although the proliferation rate was reduced when compared to saline-treated mice. However, serum levels of IFNγ, a marker cytokine for Th1 polarization, were not reduced in CPM-treated mice (see figure B above). Taken together, it appears that although lymphocyte proliferation is affected by CPM, this does not impact on IFNγ production. Together with the minor role of lymphocytes in *Apoe*$^{-/-}$ mouse models of atherosclerosis, the consistency of CPM treatment in all groups, and the transfer of native lymphocytes upon WBC reconstitution, we believe that the effect of CPM on lymphocytes is of negligible importance in this study.

Does cyclophosphamide have a general effect on collagen? All cyclophosphamide treated animals had lower levels of collagen regardless of the monocyte population reconstituted (Fig 1G).
Reply:

The direct effect of cyclophosphamide on collagen synthesis is well documented (*e.g.* Hansen & Lorenzen, Acta Pharmacol Toxicol, 1977) and we refer to this work in the revised version of the manuscript. Despite the decreased collagen synthesis in cyclophosphamide-treated mice, it is interesting to see that individual monocyte populations do not further affect local collagen metabolism.

Why is apoptosis greater in the CM depleted lesions (fig 1F)?
Reply:
This is a legitimate question raised by the referees. Classical monocytes exhibit a higher capacity to phagocytose bacteria, nanoparticles, as well as apoptotic cells (Settles *et al.,* PLoS one, 2011; Wildgruber *et al.,* PLoS one, 2009, Nahrendorf *et al.,* J Exp Med, 2007, Grage-Griebenow *et al.,* Immunobiology, 2000) when compared to non-classical monocytes. Hence, the accumulation of apoptotic cells in lesions of mice receiving WBC depleted of classical monocytes likely reflects the lack of monocytic cells with higher phagocytic capacity. We have incorporated this explanation into the result section (page 6).

With respect to the experiments in fig 3, only one time point is reported in the effects of chemokine knockouts on CM amounts is lesions. How long do CM persist in lesions before maturation to macrophages, and could the genetic manipulations alter this rate, such that a kinetic analysis would be more informative than a single time point?
Reply:
In response to this valid comment of the referee, we have now added a table displaying data of mice fed a high-fat diet for 4 weeks in the revised manuscript (new Supporting Information Table 4). These data largely corroborate the data obtained in mice receiving high-fat diet for 8 weeks, which form the foundation of this manuscript. Whereas macrophage accumulation was more markedly reduced in $Apoe^{-/-}Cx_3cr1^{-/-}$ mice than in $Apoe^{-/-}Ccr2^{-/-}$ and $Apoe^{-/-}Ccr5^{-/-}$ mice at both time points, consistent with a role of CX_3CR1 in macrophage survival (*e.g.* Landsman *et al.*, Blood, 2009), the absence of CCR1 limited macrophage accumulation at early time points but appeared to favour macrophage accumulation at later stages (page 9). In the revised manuscript, we have also emphasized that further experimentation is needed to address the role of chemokines in the processes subsequent to arterial monocyte infiltration (page 15).

We would further like to point out that figures 3 and 4 exclusively focus on the interface of monocyte transition from the blood stream to the arterial wall. Any subsequent step is subject to multiple complex influences involving maturation, survival, polarization, and egress. We do not believe that the complexity of the post-infiltration cascade can be assessed by correlative data or by assessment of monocyte/macrophage ratios at different time points.

The authors are quite aware of what they call "stage-dependent" effects of interventions on atherosclerosis. In view of this important issue, they should try to temper throughout this manuscript the strength of their conclusions that are based on study of only one time point.
Reply:
In conjunction with the new Supporting Information Table 4, we have now integrated discussions regarding stage-dependent effects at various places of the manuscript.

The authors should omit the speculation regarding targeting of HDL on page 11. This is unjustified given the current state of knowledge.
Reply:
In accordance with the referee's comment, we have now omitted our statement regarding HDL targeting.

The authors should use a better term than "atherosclerotic endothelium" on page 10.)
Reply:
We have now changed this phrase to "activated endothelium covering atherosclerotic lesions".

The use of non-standard abbreviations is not helpful to the reader. The field is already confused by use of Gr and Ly6 nomenclature. Is the use of CM and NCM here needed?
Reply:
We agree with the reviewer and hence replaced CM and NCM by the terms classical and non-classical monocytes throughout the manuscript. These terms were previously recommended to be used as standard terms (Ziegler-Heitbrock L *et al.,* Blood, 2010).

Referee #2:

1. The first conclusion is that classical monocytes are atherogenic. This is the basis of Figure 1. The conclusion recapitulates previous studies with a new, tour-de-force technique. In fact, it's remarkable that the authors see such effects on disease simply by adoptively transferring monocytes. However, it is unclear from Figure 1 whether the effect has anything to do with the accumulation of monocytes. The authors show in Supporting Figure 8 that adoptively transferred monocytes are indeed found in recipient blood. Do you also see adoptively transferred monocytes and macrophages in aorta? Given the data in Figure 1, lesional macrophages in group II should be CD45.2 and not CD45.1.
Reply:
To address this very important point raised by the referee, we have now repeated the experiments outlined for group II in figure 1 with CD45.1 recipient mice and CD45.2 donor leukocytes. To assess the presence of CD45.2 cells in the aorta, we stained aortic root sections for CD45.2 and CD45.1 and assessed the presence of $CD45.1^+$ and $CD45.2^+$ leukocytes in aortas by flow cytometry. In both analyses, we could detect CD45.2 donor-derived leukocyte in abundant numbers. These data are now incorporated as new Supporting Information Figure 3.

2. Next, the authors conclude on the basis of Figure 2 that CXCR2-CXCL1 axis is crucial to mobilize monocytes during HFD. The authors argue in Supplemental Figures 2 and 3 that the other chemokines typically associated with monocyte recruitment in atheorosclerosis are dispensable to hypercholesterolemia-induced atherosclerosis. The data are interesting. Is there statistical significance in G between HFD-isotype and HFD-anti-CXCL1? There should be if we are to conclude that CXCL1 is important. The increase shown by flow cytometry of CXCR2 on classical monocytes is modest. The authors should substantiate the finding with another method. Also, is there any impact on atherosclerosis with repeated anti-CXCL1?
Reply:
Stimulated by this interesting and highly relevant array of questions, we have now initiated an additional set of experiments, where we have further dissected the role of CXCL1 in hypercholesterolemia-induced monocytosis and subsequent lesion formation. *Apoe*$^{-/-}$ mice were fed a high-fat diet for 4 weeks, during which they received an anti-CXCL1 or an isotype control antibody. While mice injected with the isotype-control antibody developed a classical monocytosis, mice receiving an anti-CXCL1 antibody did not (new Figure 2G). In line, classical monocytes in the bone marrow and spleen of mice injected with the antibody directed against CXCL1 exhibited a trend towards increased classical monocyte counts (new Supporting Information Figure 8), indicating a retention of classical monocytes at these two sites of monocyte production. Aortic root lesion sizes of mice treated with anti-CXCL1 were smaller, when compared to mice receiving the isotype control IgG and further displayed reduced accumulation of classical monocytes as well as macrophages in the aorta as was assessed by flow cytometry (new Figure 2H/I). Data from our chemokine receptor PCR array further indicated that CXCR2 expression on classical monocytes is indeed increased under conditions of hypercholesterolemia thus confirming our flow cytometry analyses. However, we must agree with the referee that functional significance thereof is not clear and we hence moved these data into the supplementary information (Supporting Information Figure 7).

3. Finally, the authors show that CCR1 and CCR5 but not CCR2 or CX3CR1 are involved in monocyte accumulation to lesions. This is the most controversial part of the paper and probably the most important. It must therefore be very convincing - unfortunately, it is not. First, the authors

should report lesion size and number of macrophages in CCR1 and CCR5 apoE mice. In figure 3 the authors show reduced numbers of monocytes in the aorta in CCR1 and CCR5 mice which suggests a problem with influx but could also mean maturation, survival, exit. Second, the CFSE experiment is not convincing. There are almost no CFSE cells accumulating in lesions so even with the stars that denote statistical significance in Figure 4B the data are weak. The authors should perform experiments to more convincingly show that CCR1 and CCR5 are the essential chemokine receptors.
Reply:
In light of previous publications in the field, we certainly agree with the reviewer that the information provided in figures 3 and 4 maybe somewhat controversial and hence requires corroboration. However, we would like to point out, that figure 4 is a consequence of figure 3, the latter displaying a lack of correlation between circulating and lesional classical monocytes. As this could indicate a defect in recruitment as well as alterations in maturation or egress, we performed experiments detailed in figure 4. Hence, we designed two alternative strategies that allow to specifically address the interface of monocyte transition from the blood stream to the arterial wall independently of homeostatic or post-emigration processes. To our knowledge, apart from these three approaches (correlation studies, intravital microscopy using short term treatment with inhibitors, adoptive transfer experiments with short circulation time post transfer) employed here, there is no additional experimental setup that allows to specifically investigate infiltration of classical monocytes independently of homeostatic and post-recruitment mechanisms only. Even murine parasymbiosis models, which are for ethical concerns impossible to perform in Europe, have their limitations. In these setups, the accumulation of monocytes in arterial lesions over several weeks is subject to influences by many mechanisms of monocyte differentiation, polarization, maturation, and egress and hence no clear-cut conclusion on emigration can be drawn.

To further corroborate the data provided in figures 3 and 4, we have now added an extensive table summarizing lesion sizes, circulating monocyte counts, circulating classical monocyte counts, prevalence of classical monocytes and macrophages in the aorta, as well as the correlation of circulating and lesional classical monocytes. All these parameters are provided for *Apoe*$^{-/-}$, *Apoe*$^{-/-}$*Ccr1*$^{-/-}$, *Apoe*$^{-/-}$*Ccr2*$^{-/-}$, *Apoe*$^{-/-}$*Ccr5*$^{-/-}$, and *Apoe*$^{-/-}$*Cx*$_3$*cr1*$^{-/-}$ at two different time points of high-fat diet feeding (new Supporting Information Table 4).

To further substantiate data from the adoptive transfer experiments, we employed the same strategy but instead used the CD45.1/CD45.2 system to track classical monocytes. Based on improved discrimination of donor cells within the aortas of CD45.1/*Ldlr*$^{-/-}$ mice, we can corroborate both the number of lesional monocytes as well as the importance of CCR1 and CCR5 for arterial monocyte influx (new Figure 4C/D). The numbers of donor-derived lesional monocytes in both adoptive transfer approaches employed in this study are in the range of what was found in previous studies using similar approaches (*e.g.* Tacke *et al.*, J Clin Invest, 2007) and may hence truly reflect monocyte recruitment rates. Thus, we believe that further studies are required to dissect rates of arterial monocyte turn-over.

Referee #3:

Before acceptance for publication it is therefore suggested to
- include references to decoy receptors such as D6 and discussions thereof
Reply:
As suggested by the referee, we have made reference to decoy receptors in the discussion section (page 14).

- include references to glycosaminoglycan (GAG) and proteoglycan chemokine co-receptors such as heparin sulfate and discussions thereof
Reply:
As suggested by the referee, we have included references to GAGs and chemokine co-receptors in the discussion section (page 14/15).

- investigate (e.g. by real time PCR or by FACS analyses) the involvement of the above-mentioned non-signalling chemokine co-receptors
Reply:
Various studies will be required to fully investigate and understand the role of non-signaling chemokine co-receptors in atherosclerosis. We thank the reviewer for giving us the chance to provide initial data on the role of such receptors in atherosclerosis. Here, we investigate the expression of decoy receptors D6 and CXCR7 and the CCL5 co-receptor CD44 on classical monocytes by flow cytometry. In these experiments we could not find increased expression under conditions of hypercholesterolemia (new Supporting Information Figure 10).

Since many chemokine-targeting therapies have failed for various reasons in the past, it is required to aim at a fairly complete picture of chemokines/receptor/co-receptor networks before seriously speculating about therapeutic targeting of CCL5/CCR1/CCR5 interactions.
Reply:
We fully agree with the referee on this point. We have therefore removed speculations about possible therapeutic targeting of the CCL5-CCR1/-CCR5 axis.

2nd Editorial Decision 17 December 2012

Thank you for the submission of your revised manuscript to EMBO Molecular Medicine. We have now received the enclosed reports from the referees that were asked to re-assess it. As you will see the reviewers are now globally supportive and I am pleased to inform you that we will be able to accept your manuscript pending the following final amendments:

1) Please make sure you modify your Abstract and Discussion as suggested by Reviewer 1.
2) The text in the figures is rather blocky/blurry. Please provide higher resolution versions, and check to make sure that text/line-art remains clear even when zooming in.
3) Where you have not done so, please follow the other instructions listed below

I strongly advise you to submit your revised manuscript within two days to ensure, provided the changes have been satisfactorily applied, acceptance before the Holiday season.

I look forward to seeing a revised form of your manuscript as soon as possible.

***** Reviewer's comments *****

Referee #1 (General Remarks):

The authors have responded appropriately to my major concerns, and provided relevant additional new experimental data. I suggest that they remove the modifier "unequivocally" before "establish" in the abstract, and last paragraph of thje discussion as redundant, and unjustified given the contrived nature of their model. I also think their dismissal of T-cells in atherosclerosis on the basis of the Dansky paper ignores a large body of other data regarding modulatory effects of T cells in atherogenesis.

Referee #2 (Comments on Novelty/Model System):

This is a very strong revision. The authors have addressed all my questions very well.

Referee #2 (General Remarks):

I have no more remarks.

Referee #3 (Comments on Novelty/Model System):

The data in the manuscript are highly relevant to the specialised field of the authors. It would, however, be very interesting to see whether similar mobilisation and recruitment mechanisms could be responsible for other monocytic-related disorders.

Referee #3 (Remarks):

The concerns and the suggestions of this reviewer have been met by the authors in their revised manuscript.

2nd Revision - authors' response 19 December 2012

As suggested by reviewer #1 we have deleted "unequivocally" in abstract and discussion. With regard to the paper of Dansky et al. since our study was performed with mice receiving high-fat diet, we believe that no additional comment was required, but we can certainly discuss this further if you request so.

© EMBO
