# Supplementary material for: Distinct functions of chemokine receptor axes in the atherogenic mobilization and recruitment of classical monocytes
Source: EMBO Mol Med. 2013 Feb 18;5(3):471–81. doi: 10.1002/emmm.201201717 (PMC3598085; doi:10.1002/emmm.201201717)
Supplement: Supplementary file 2 [file emmm0005-0471-SD2.pdf]

Supporting information

Supporting Information Figure 1: Antibody-based monocyte selection does not activate leukocytes. 2

Supporting Information Figure 2: Validation of white blood cell reconstitution. 3

Supporting Information Figure 3: Adoptively transferred leukocytes accumulate in atherosclerotic lesions. 4

Supporting Information Figure 4: Apoptotic cells are partially of macrophage origin. 5

Supporting Information Figure 5: CCL2/CCL7 serum levels are unaltered by hypercholesterolemia. 6

Supporting Information Figure 6: Hypercholesterolemia-induced monocytosis is independent of CCR1, CCR2, CCR5, and CX_3CR1. 7

Supporting Information Figure 7: Effect of hypercholesterolemia on chemokine receptor surface expression on classical monocytes. 8

Supporting Information Figure 8: Effect of CXCL1-neutralization on classical monocytes in bone marrow and spleen. 9

Supporting Information Figure 9: Plasma CXCL1 levels are increased in patients with moderate hypercholesterolemia. 10

Supporting Information Figure 10: High-fat diet does not alter expression of D6, CXCR7, or CD44 on classical monocytes. 11

Supporting Information Figure 11: Gating strategy for detection of classical monocytes in aortas. 12

Supporting Information Figure 12: Aortic recruitment of adoptively transferred classical monocytes depends on CCR1 and CCR5. 13

Supporting Information Table 1: Repeated cyclophosphamide-injection induces severe leukopenia. 14

Supporting Information Table 2: Leukocyte ablation and reconstitution does not alter blood lipid levels. 15

Supporting Information Table 3: Leukocyte ablation and reconstitution does not alter body or spleen weights. 16

Supporting Information Table 4: Accumulation of atherosclerotic lesion sizes, monocytic cell counts in the blood and in the aorta, and correlation thereof after 4 and 8 weeks of high-fat diet. 17

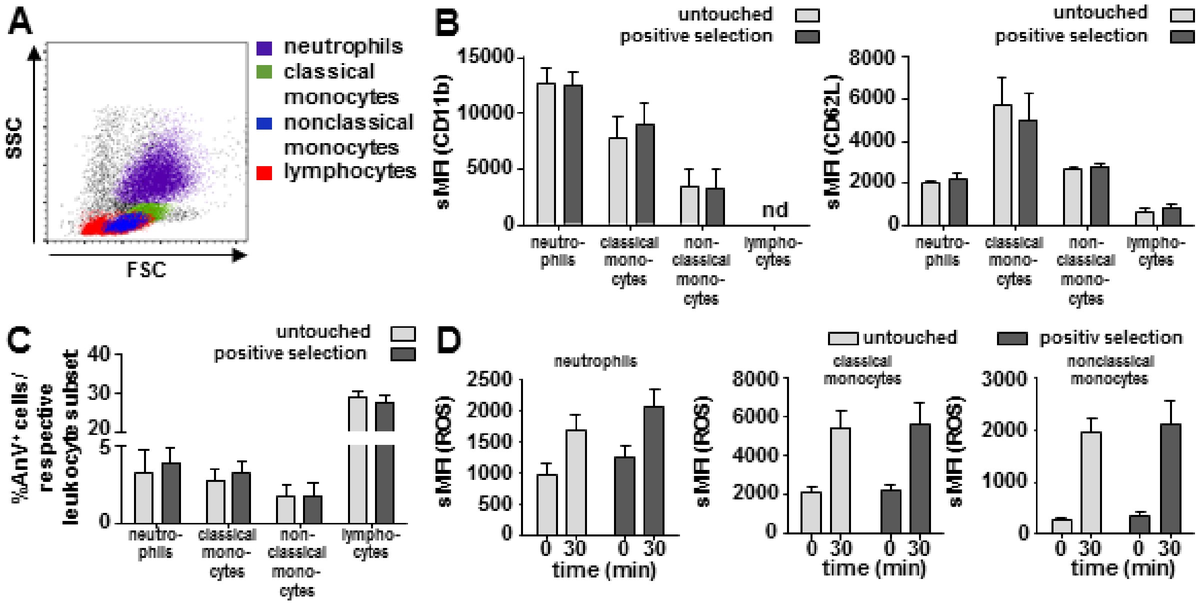

Supporting Information Figure 1: Antibody-based monocyte selection does not activate leukocytes. Leukocytes were incubated with an antibody cocktail (anti-CD45, anti-CD115, anti-Gr1, referred to as ‘positive selection’) or left untreated (referred to as ‘untouched’) and white blood cells were subsequently FACS-sorted. **A:** Display of FSC vs. SSC of leukocyte subpopulations after phenotypic identification based on SSC, anti-CD11b, anti-Ly6C, and anti-CD3 staining. **B:** Analysis of CD11b (left) and CD62L (right) surface expression on leukocyte subsets. **C:** Apoptosis analysis as assessed by Annexin V binding properties. **D:** Reactive oxygen species production in myeloid cell subsets in response to PMA (20 nM). All data are expressed as mean ± SD. n = 6 for each group (Students t-test).

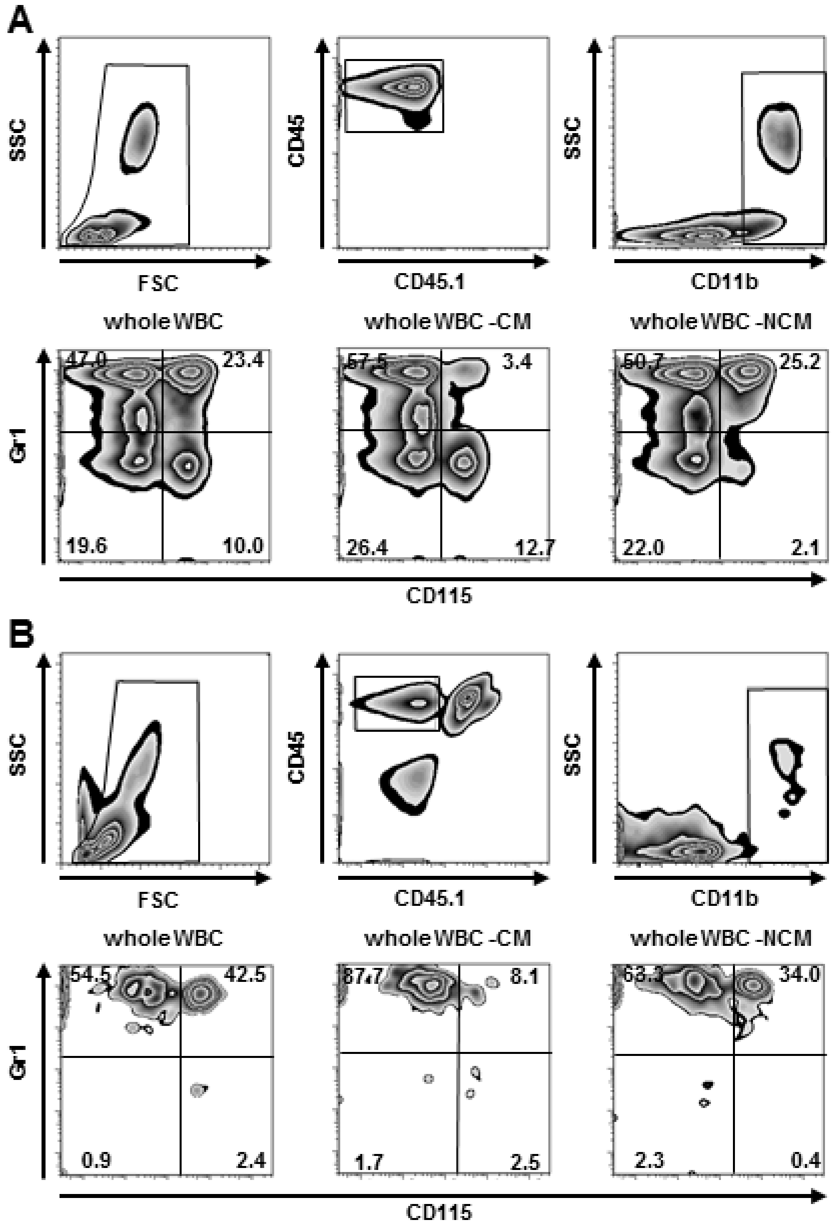

Supporting Information Figure 2: Validation of white blood cell reconstitution. A: Efficiency of FACS-sorted depletion of monocyte subpopulations. White blood cells from CD45.2 mice were harvested (top row) and left intact (bottom left), depleted of classical (bottom middle), or depleted of non-classical monocytes (bottom right). **B:** Reconstitution of myeloid cell subsets. Identification of $CD45.1^{-}CD45.2^{+}CD11b^{+}$ donor myeloid cells in recipient mice (top row). Recipient mice were injected with white blood cells (bottom left), or white blood cells depleted of classical monocytes (bottom middle) or depleted of non-classical monocytes (bottom right). Frequencies of $CD45.1^{-}CD45.2^{+}CD11b^{+}$ parent gate are displayed for each quadrant.

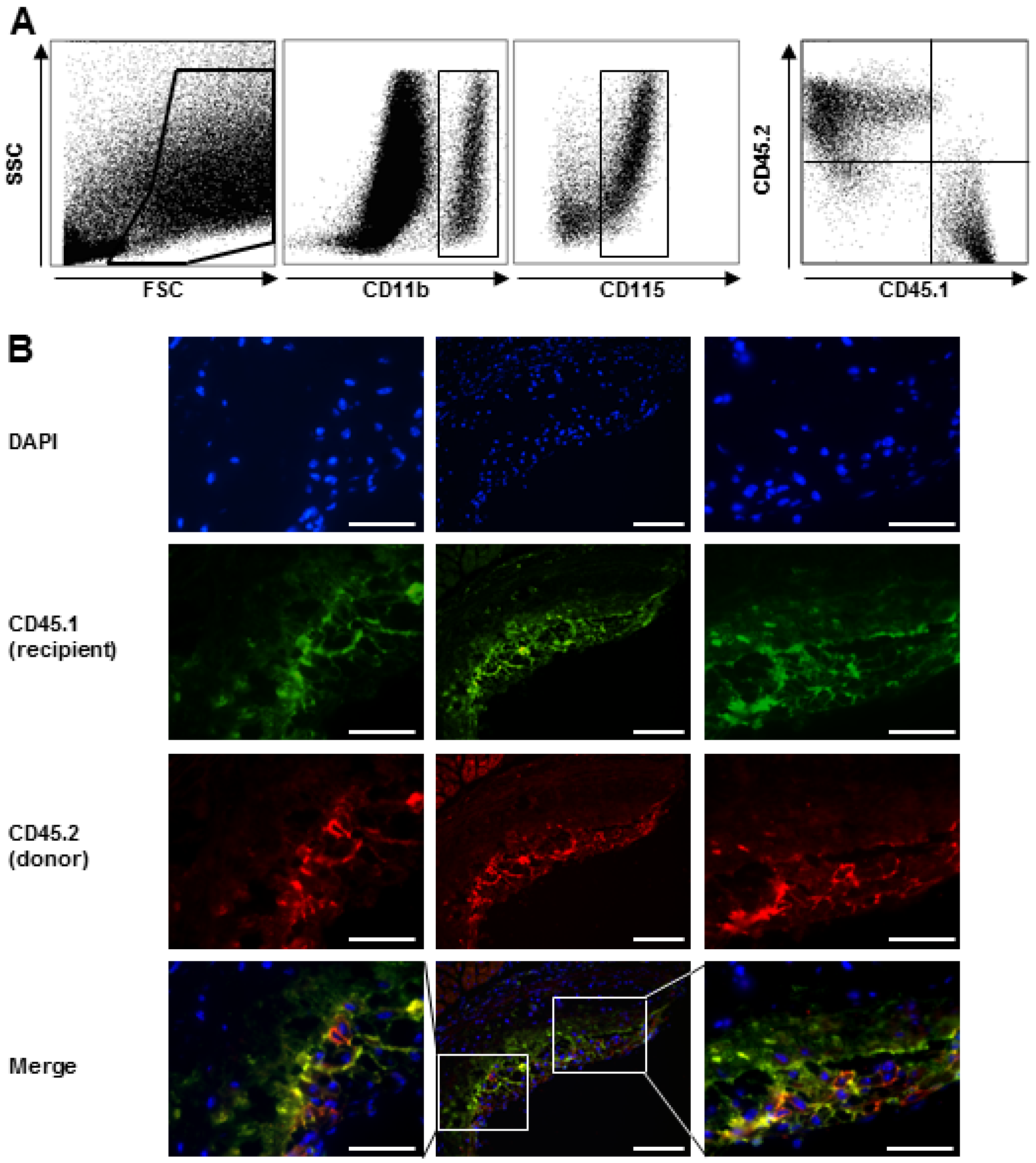

Supporting Information Figure 3: Adoptively transferred leukocytes accumulate in atherosclerotic lesions. Male CD45.1 *LDLr*$^{-/-}$ mice 6 weeks of age were fed a high-fat diet (HFD) for a total of 8 weeks. After 4 weeks of HFD mice were treated with cyclophosphamide (CPM) 2x/week. Mice were reconstituted with leukocytes from CD45.2 *Apoe*$^{-/-}$ donor mice by i.v. injections 2x/week using one donor mouse/recipient each 1 day after CPM treatment. **A:** Representative FACS charts displaying accumulation of CD45.2 monocytic cells in the aortas of CD45.1 *LDLr*$^{-/-}$ mice. **B:** CD45.1- and CD45.2-positive leukocytes were identified in aortic root sections of recipient mice. Scale bar indicates 50µm left/right group of pictures and 100µm in the central group.

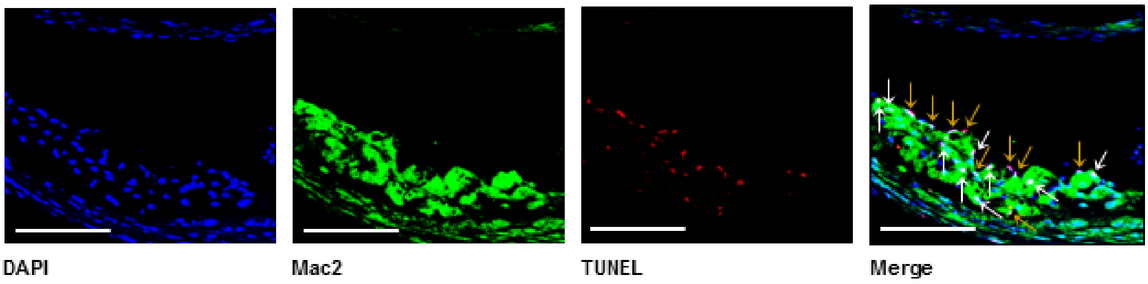

Supporting Information Figure 4: Apoptotic cells are partially of macrophage origin. Aortic root sections were stained with TUNEL and anti-Mac2 antibodies. Cells positive for TUNEL and Mac2, white arrows; cells positive for TUNEL only, brown arrows. Scale bar is 50 µm.

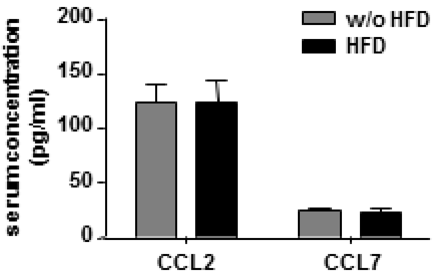

Supporting Information Figure 5: CCL2/CCL7 serum levels are unaltered by hypercholesterolemia. Concentrations of serum CCL2 and CCL7 of *Apoe*$^{-/-}$ mice fed a chow diet or high-fat diet (HFD) for 8 weeks were quantified by ELISA. All data are expressed as mean ± SD. n = 8-11 for each group (Students t-test).

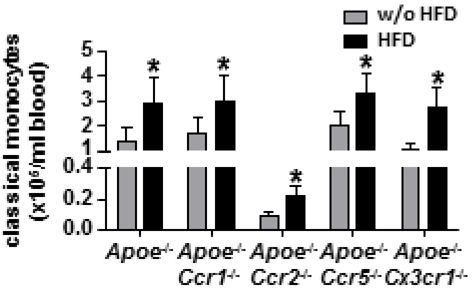

Supporting Information Figure 6: Hypercholesterolemia-induced monocytosis is independent of CCR1, CCR2, CCR5, and CX_3CR1. Classical monocytes were quantified in indicated mouse strains fed a chow diet or high-fat diet (HFD) for 8 weeks. All data are expressed as mean ± SD. * denotes significant differences between groups. n = 8-9 for each group (Students t-test).

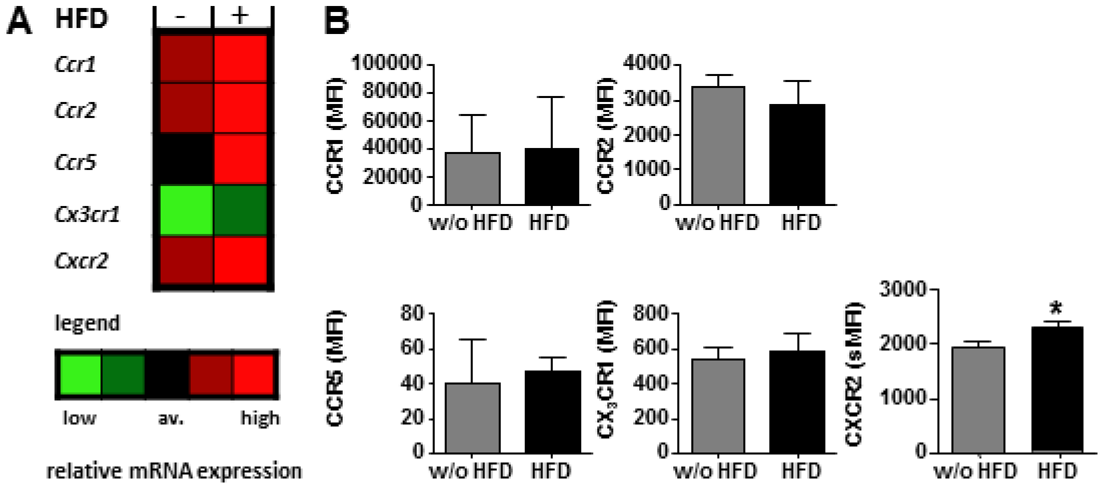

Supporting Information Figure 7: Effect of hypercholesterolemia on chemokine receptor surface expression on classical monocytes. A: Transcriptional expression changes of indicated chemokine receptors in classical monocytes of *Apoe*$^{-/-}$ mice fed a chow diet or high-fat diet (HFD) for 8 weeks were assessed by PCR array studies. **B:** Expression of CCR1, CCR2, CCR5, CX_3CR1, and CXCR2 (mean fluorescence intensity, MFI) on classical monocytes from *Apoe*$^{-/-}$ mice fed normal chow or HFD for 8 weeks were assessed by FACS. All data are expressed as mean ± SD. * indicates significant difference between groups. n = 3-5 for each group (Mann-Whitney U-test).

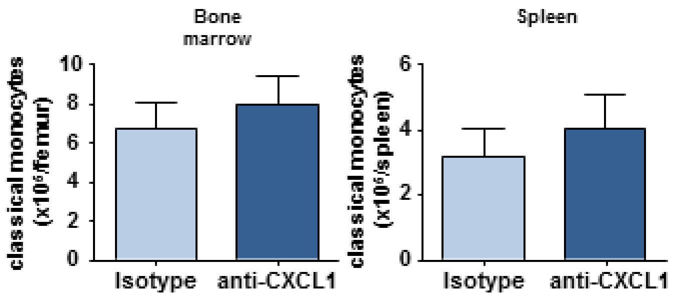

-

Supporting Information Figure 8: Effect of CXCL1-neutralization on classical monocytes in bone marrow and spleen. *Apoe*$^{-/-}$ mice were fed high fat diet for 4 weeks and injected (5µg i.p., daily during first week, 3x/week in subsequent weeks) with isotype control or an antibody to mCXCL1. Displayed are absolute counts of classical monocytes in the bone marrow (left) or spleen (right), as assessed by flow cytometry. All data are expressed as mean±SD. n=8 for each group

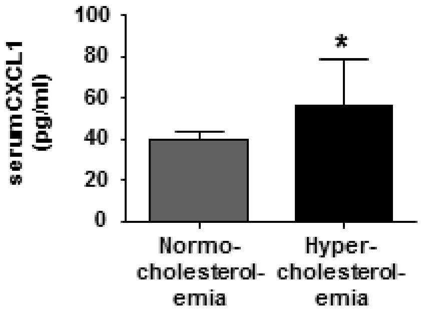

Supporting Information Figure 9: Plasma CXCL1 levels are increased in patients with moderate hypercholesterolemia. Plasma CXCL1 was assessed in patients with moderate hypercholesterolemia by ELISA. n = 10 for each bar (Students t-test). * denotes significant differences between groups.

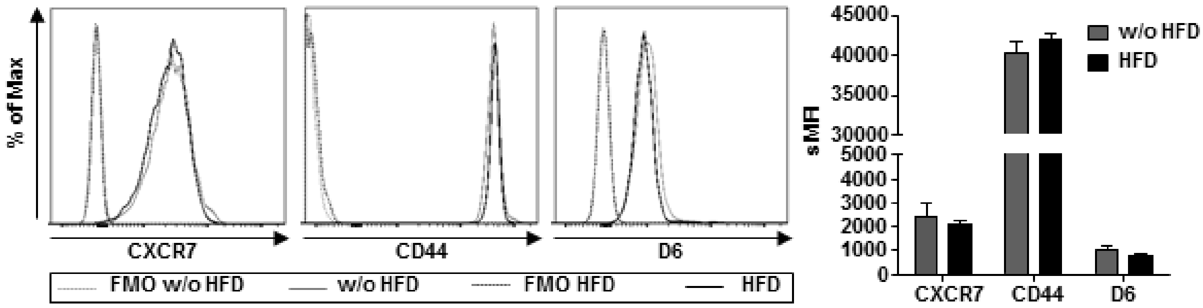

Supporting Information Figure 10: High-fat diet does not alter expression of D6, CXCR7, or CD44 on classical monocytes. *Apoe*$^{-/-}$ mice were fed a high-fat diet (HFD) for 4 weeks or received chow diet. Expression of CXCR7, CD44, and D6 on classical monocytes was measured by flow cytometry. Displayed are representative histograms and quantification. All data are expressed as mean ± SD. n = 4 for each group. FMO, fluorescence minus one; sMFI, specific mean fluorescence intensity.

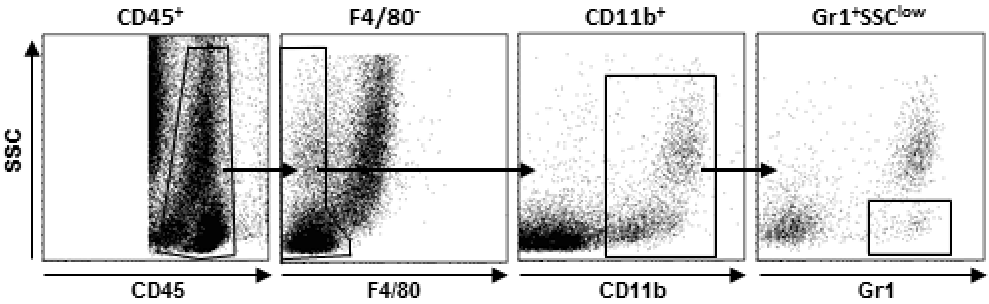

Supporting Information Figure 11: Gating strategy for detection of classical monocytes in aortas. Shown are representative dot blots and gates.

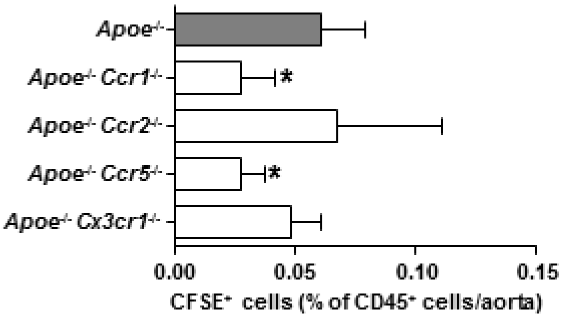

Supporting Information Figure 12: Aortic recruitment of adoptively transferred classical monocytes depends on CCR1 and CCR5. Classical monocytes (10^6) isolated from the bone marrow of indicated donor mouse strains by FACS sorting were injected into *Apoe*$^{-/-}$ recipients after labeling with the cell tracker CFSE and allowed to circulate for 24 hours. Both donor mice and recipients had been on high-fat diet (HFD) for 8 weeks. Number of CFSE^{+} cells in the aorta is expressed in % of CD45^{+} cells. * denotes significant differences compared to injection of classical monocytes from *Apoe*$^{-/-}$ donor ice. n = 7 for each group (Kruskal-Wallis with Dunns post-hoc test).

Tables

	granulocytes	monocytes	classical monocytes	nonclassical monocytes	T-cells	B-cells
**control**	7.3 x 10^5 +/- 1.5 x 10^5	2.5 x 10^5 +/- 0.4 x 10^5	1.8 x 10^5 +/- 0.4 x 10^5	0.7 x 10^5 +/- 0.1 x 10^5	19.7 x 10^5 +/- 3.1 x 10^5	37.2 x 10^5 +/- 8.0 x 10^5
**CPM**	0.2 x 10^5* +/- 0.1 x 10^5	0.01 x 10^5* +/- 0.1 x 10^5	0.1 x 10^5* +/- 0.1 x 10^5	0.1 x 10^5* +/- 0.1 x 10^5	7.1 x 10^5* +/- 1.3 x 10^5	0.7 x 10^5* +/- 0.4 x 10^5

Supporting Information Table 1: Repeated cyclophosphamide-injection induces severe leukopenia. Injection of 100 mg/kg cyclophosphamide (CPM) two times a week leads to significantly reduced circulating leukocyte subpopulations. * indicates significant difference compared to respective controls. n = 6 for each group (Mann-Whitney U-test).

	Triglycerides	Total Cholesterol	LDL/VLDL	HDL
Group 0 (HFD only)	179.1 +/- 57.9	539.9 +/- 59.7	264.4 +/- 77.8	189.8 +/- 74.9
Group I (HFD + CPM injection)	184.3 +/- 54.17	516.3 +/- 69.02	238.9 +/- 52.3	158.4 +/- 74.4
Group II (HFD + CPM injection + reconstituted with WBC)	201.1 +/- 40.8	490.8 +/- 58.3	180.3 +/- 68.1	142.0 +/- 29.1
Group III (HFD + CPM injection + reconstituted with WBC – classical monocytes)	208.5 +/- 56.17	489.4 +/- 80.4	218.8 +/- 74.2	131.5 +/- 30.1
Group IV (HFD + CPM injection + reconstituted with WBC – non-classical monocytes)	188.0 +/- 68.6	506.7 +/- 89.03	198.8 +/- 87.9	169.0 +/- 72.77

Supporting Information Table 2: Leukocyte ablation and reconstitution does not alter blood lipid levels. Plasma lipid levels were assessed at the end of the ablation-reconstitution cycle by use of EnzyChrom™ Triglyceride Assay Kit and the EnzyChrom™ HDL and LDL/VLDL Assay Kit. n = 9-12 for each group.

	Body weight (in g)	Spleen weight (in mg)
Before HFD	16.6 +/- 0.1	N.D.
Group 0 (HFD only)	30.2 +/- 2.0	71.5 +/- 10.9
Group I (HFD + CPM injection)	27.4 +/- 2.1	84.8 +/- 22.7
Group II (HFD + CPM injection + reconstituted with WBC)	27.6 +/- 1.1	105.0 +/- 25.5
Group III (HFD + CPM injection + reconstituted with WBC – classical monocytes)	28.00 +/- 1.9	108.0 +/- 15.7
Group IV (HFD + CPM injection + reconstituted with WBC – non-classical monocytes)	27.2 +/- 2.0	111.9 +/- 14.7

Supporting Information Table 3: Leukocyte ablation and reconstitution does not alter body or spleen weights. Body and spleen weights were assessed before initiating high-fat diet as well as at the end of the experiment in each group**.** n = 9-12 for each group.

	Geno-type	Oil-red O+ area (%of aortic root area)	total circulating monocyte count (x10^5/ml)	circulating CM count (x10^5/ml)	CM number/ aorta	Correlation circulating CM/aortic CM	Macrophage number/ aorta
4 weeks HFD	*Apoe*$^{-/-}$	8.4 +/- 1.9	5.8 +/- 1.7	3.7 +/- 1.2	187.5 +/- 47.5	**r = 0.8855 p = 0.0457**	17723 +/- 3199
	Apoe$^{-/-}$ *Ccr1*$^{-/-}$	**3.8*** +/- 1.2	5.8 +/- 2.2	4.3 +/- 1.7	**100.3*** +/- 18.8	r = -0.6426 p = 0.5557	**4113*** +/- 1095
	Apoe$^{-/-}$ *Ccr2*$^{-/-}$	**3.3*** +/- 1.9	**1.2*** +/- 0.2	**0.4*** +/- 0.1	**84.2*** +/- 45.5	**r = 0.9570 p = 0.0430**	**8230*** +/- 2906
	Apoe$^{-/-}$ *Ccr5*$^{-/-}$	**3.6*** +/- 1.2	6.5 +/- 1.2	4.4 +/- 0.8	**114.0*** +/- 13.6	r = 0.0109 p = 0.9890	**9554*** +/- 3063
	Apoe$^{-/-}$ *Cx$_3$cr1*$^{-/-}$	**2.8*** +/- 1.8	4.5 +/- 2.0	3.4 +/- 1.5	162.0 +/- 92.9	**r = 0.9152 p = 0.0293**	**4567*** +/- 2735
8 weeks HFD	*Apoe*$^{-/-}$	12.0 +/- 3.1	4.9 +/- 2.1	3.0 +/- 1.2	178.8 +/- 74.6	**r = 0.8277 p = 0.0001**	18738 +/- 2933
	Apoe$^{-/-}$ *Ccr1*$^{-/-}$	9.6 +/- 2.2	4.6 +/- 2.0	2.8 +/- 1.1	**83.9*** +/- 33.1	r = 0.0914 p = 0.7460	14909 +/- 2939
	Apoe$^{-/-}$ *Ccr2*$^{-/-}$	**5.5*** +/- 2.4	**0.9*** +/- 0.5	**0.3*** +/- 0.2	**48.2*** +/- 22.9	**r = 0.8885 p = 0.0001**	**11551*** +/- 3317
	Apoe$^{-/-}$ *Ccr5*$^{-/-}$	**6.6*** +/- 2.7	4.0 +/- 1.7	2.9 +/- 1.2	**102.4*** +/- 30.5	r = -0.1003 p = 0.7221	**10389*** +/- 3572
	Apoe$^{-/-}$ *Cx$_3$cr1*$^{-/-}$	**4.4*** +/- 2.5	3.5 +/- 0.8	2.7 +/- 0.8	148.1 +/- 56.19	**r = 0.8048 p = 0.0003**	**7542*** +/- 2005

Supporting Information Table 4: Accumulation of atherosclerotic lesion sizes, monocytic cell counts in the blood and in the aorta, and correlation thereof after 4 and 8 weeks of HFD. All data are expressed as mean ± SD. n = 5-7 for mice fed a HFD for 4 weeks and n = 13-15 for mice fed a HFD for 8 weeks. * indicates significant difference compared to respective *Apoe*$^{-/-}$ mice. HFD, high-fat diet; CM, classical monocytes; NCM, non-classical monocytes.
